# Potassium response and homeostasis in *Mycobacterium tuberculosis* modulates environmental adaptation and is important for host colonization

**DOI:** 10.1371/journal.ppat.1007591

**Published:** 2019-02-04

**Authors:** Nathan J. MacGilvary, Yuzo L. Kevorkian, Shumin Tan

**Affiliations:** 1 Department of Molecular Biology and Microbiology, Tufts University School of Medicine, Boston, Massachusetts, United States of America; 2 Graduate Program in Molecular Microbiology, Sackler School of Graduate Biomedical Sciences, Tufts University, Boston, Massachusetts, United States of America; National Institutes of Health, UNITED STATES

## Abstract

Successful host colonization by bacteria requires sensing and response to the local ionic milieu, and coordination of responses with the maintenance of ionic homeostasis in the face of changing conditions. We previously discovered that *Mycobacterium tuberculosis* (Mtb) responds synergistically to chloride (Cl^-^) and pH, as cues to the immune status of its host. This raised the intriguing concept of abundant ions as important environmental signals, and we have now uncovered potassium (K^+^) as an ion that can significantly impact colonization by Mtb. The bacterium has a unique transcriptional response to changes in environmental K^+^ levels, with both distinct and shared regulatory mechanisms controlling Mtb response to the ionic signals of K^+^, Cl^-^, and pH. We demonstrate that intraphagosomal K^+^ levels increase during macrophage phagosome maturation, and find using a novel fluorescent K^+^-responsive reporter Mtb strain that K^+^ is not limiting during macrophage infection. Disruption of Mtb K^+^ homeostasis by deletion of the Trk K^+^ uptake system results in dampening of the bacterial response to pH and Cl^-^, and attenuation in host colonization, both in primary murine bone marrow-derived macrophages and *in vivo* in a murine model of Mtb infection. Our study reveals how bacterial ionic homeostasis can impact environmental ionic responses, and highlights the important role that abundant ions can play during host colonization by Mtb.

## Author summary

Tuberculosis is the leading cause of death from infectious diseases globally, and knowledge of the fundamental biology underlying successful host colonization by *Mycobacterium tuberculosis* (Mtb) is critical for understanding its pathogenicity. Our study focuses on one such important facet—the ability of Mtb to sense and respond to changing environmental ionic signals, and its relation to maintenance of bacterial ionic homeostasis. Potassium (K^+^) is the most abundant ion in both mammalian and bacterial cells, but much remains unknown about how it may affect host-pathogen interactions. We show here that Mtb has distinct gene expression changes in response to variation in environmental K^+^ levels. Importantly, disruption of a Mtb K^+^ uptake system reduces the ability of Mtb to respond to other key ionic cues, and results in diminished host colonization. Our study reveals novel roles of K^+^ during Mtb-host interactions, and suggests the broader importance of abundant ions in bacterial pathogenicity.

## Introduction

Ions are a fundamental component of organisms, playing roles in myriad biological processes. The ionic milieu surrounding a bacterium during infection varies with location and the immune response [1–7], and successful host colonization thus requires proper sensing and response to the local ionic milieu, and the ability to coordinate responses with the maintenance of ionic homeostasis in the face of changing conditions. For *Mycobacterium tuberculosis* (Mtb), pH ([H^+^] flux) is well-established as a critical signal during host colonization [8, 9], with the bacteria concurrently able to robustly maintain intrabacterial pH [10]. We have since identified chloride (Cl^-^) as an ionic cue important for Mtb [2], but it remains an open question what other abundant ions may flux during Mtb colonization, and how bacterial ionic homeostasis may affect response to environmental ionic cues. Mtb is a bacterium of immense public health importance, being the leading cause of death from infectious diseases worldwide [11]. Uncovering facets of the local environment that the bacteria respond to, and how fundamental aspects of Mtb biology relate to environmental response and colonization success, is critical for comprehension of Mtb-host interactions and has the potential to reveal novel nodes that can be targeted for therapeutic purposes.

Our previous discovery of Cl^-^ as a novel environmental signal that Mtb responds to in synergy with pH indicated the significant role that flux and homeostasis of abundant ions can play during Mtb infection and disease [2]. In this context, potassium (K^+^), the most abundant intracellular cation in both mammalian and bacterial cells, stands out as a compelling candidate for study. In mammalian systems, K^+^ has fundamental roles in processes as diverse as renal function, muscle contraction, and neuronal information transmission [12–15]. There is also increasing appreciation for the importance of K^+^ in non-excitable cells, including in epithelial and immune cells in the lung [16, 17]. In contrast, studies on K^+^ in bacterial systems have predominantly focused on its role in osmoprotection [18]. However, results from a few studies have begun to hint at the broader importance of K^+^ in influencing bacterial pathogenicity and disease outcome. For example, higher environmental K^+^ concentrations ([K^+^]) increased expression of virulence factors and host cell invasion by the food-borne pathogen *Salmonella enterica* [19]. Inactivation of the Trk K^+^ uptake system in *S*. *enterica* further resulted in defects in effector protein secretion through the bacterium’s type III secretion system, and attenuation in virulence in both murine and chick models of infection [20]. A bacterial K^+^ uptake system has been shown to be essential for *Helicobacter pylori* gastric colonization [21], and K^+^ has also been shown to be part of a signal cascade that enables coordination of bacterial metabolic states in a *Bacillus subtilis* biofilm [22]. Mtb possesses two K^+^ uptake systems and one K^+^ efflux system [23–25], but the role of K^+^ in Mtb infection biology has been largely unstudied. How does Mtb respond transcriptionally to local changes in [K^+^], and how may this response be linked to the bacterial response to other environmental cues? What is the impact of perturbation of bacterial K^+^ homeostasis on successful host colonization by Mtb?

In this study, we show that Mtb has a unique transcriptional response to changes in environmental [K^+^]. While the pH and Cl^-^ response regulator PhoPR does not affect Mtb response to external K^+^, the novel transcriptional repressor Rv0500A regulates bacterial response not just to pH and Cl^-^, but also K^+^. By using a K^+^-responsive fluorescent reporter Mtb strain in macrophage infections, we find that K^+^ in the phagosome is not limiting, and instead increases during phagosome maturation. Disruption of Mtb K^+^ homeostasis by deletion of the Trk K^+^ uptake system (Δ*ceoBC*) results in dampening of the bacterial response to pH and Cl^-^, revealing the links between environmental ionic responses and bacterial ionic homeostasis. Finally, we show that the Δ*ceoBC* mutant is attenuated for host colonization, both in primary murine bone marrow-derived macrophages and *in vivo* in a murine model of Mtb infection.

## Results and discussion

### Unique Mtb gene transcription signature in response to changes in environmental K^+^ levels

**Fig 1:**
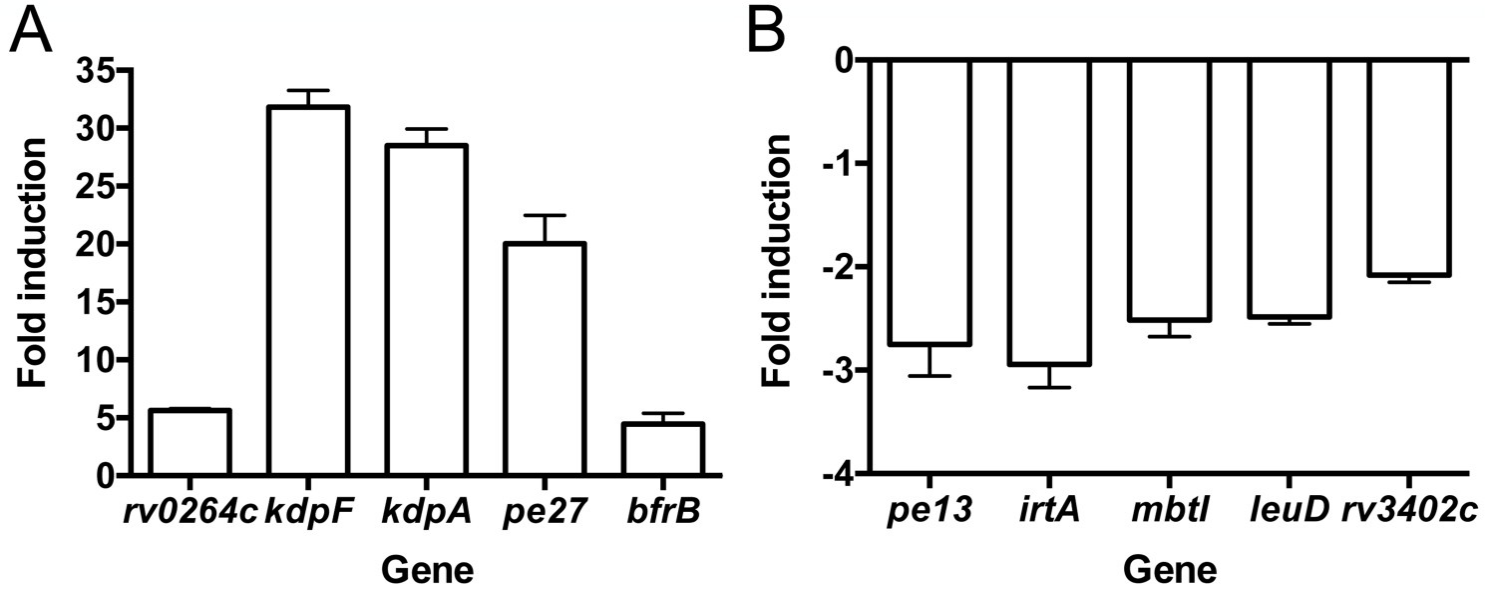
Transcriptional response of Mtb to low environmental [K^+^]. (A and B) qRT-PCR of gene expression in WT grown in K^+^-free 7H9, pH 7, compared to standard 7H9, pH 7, for 4 hours. Upregulated genes are shown in (A), and downregulated genes are shown in (B). Data are shown as means ± SD from 3 technical replicates, representative of 2–3 independent experiments.

To elucidate the global transcriptional response of Mtb to changes in environmental [K^+^], we performed RNA sequencing (RNAseq) on Mtb exposed for 4 hours to K^+^-free 7H9 media, and compared it to samples in standard 7H9 media ([K^+^] = 7.35 mM). We found that 43 genes were upregulated and 66 genes downregulated (>2-fold, p<0.05) in response to low [K^+^] (S1 and S2 Tables). The upregulated gene list represented a unique gene set, with slight overlap with genes upregulated in response to high [Cl^-^], but otherwise little overlap when compared to other known environmental regulons (Table 1) [2, 8, 26–28]. Among the strongest upregulated genes were those belonging to the *kdpFABC* operon. These genes encode the inducible K^+^ uptake ATPase KpdFABC, and their robust upregulation served to affirm their function in low environmental [K^+^] [24]. Interestingly, several genes related to iron uptake were repressed upon Mtb exposure to low environmental [K^+^] (S2 Table). These included five mycobactin genes (*mbtB*, *mbtC*, *mbtD*, *mbtI*, and *mbtK*), as well as the iron-regulated transporter genes *irtA* and *irtB*. Comparison to the previously reported gene set downregulated under conditions of high iron revealed a significant overlap (Table 1) [27]. Oxidative stress is known to induce the expression of iron uptake genes, including the *mbt* genes [26], and we accordingly also observed an overlap between the low [K^+^] downregulated gene set and genes upregulated upon exposure to oxidative stress (Table 1). Quantitative real time PCR (qRT-PCR) of candidate genes confirmed the RNAseq data (Fig 1). In addition, no expression differences in the candidate genes were observed in sodium (Na^+^)-free media, reinforcing the specificity of the K^+^ response regulon (S1 Fig).

**Table 1:** Comparison of low [K^+^] response regulon to different environmental response regulons.

*In vitro* stimulus	Genes in reference list^a^	Overlap with upregulated gene list in K^+^-free 7H9	Overlap with downregulated gene list in K^+^-free 7H9
Acid stress^c^	291	2	4
Oxidative stress^d^	142	0	21^b^
High iron^e^	66	1	19^b^
Hypoxia^f^	50	3	0
High [Cl^-^]^g^	32	5^b^	1

^a^ All reference lists refer to upregulated gene sets, except for the high iron set, which refers to the downregulated gene set.

^b^ Overlap for these comparisons are significant at p<0.01 (hypergeometric distribution).

^c^ Data from Rohde *et al*. [8]

^d^ Data from Voskuil *et al*. [26]

^e^ Data from Rodriguez *et al*. [27]

^f^ Data from Park *et al*. [28]

^g^ Data from Tan *et al*. [2]

These transcriptional data reveal a significant response of Mtb to limiting external [K^+^], with a unique signature of upregulated genes. The unexpected overlap with the iron and oxidative stress response regulons in the downregulated gene set provides an intriguing base for future studies.

### *kdpF’*::GFP functions as a specific reporter of Mtb response to environmental K^+^ levels

**Fig 2:**
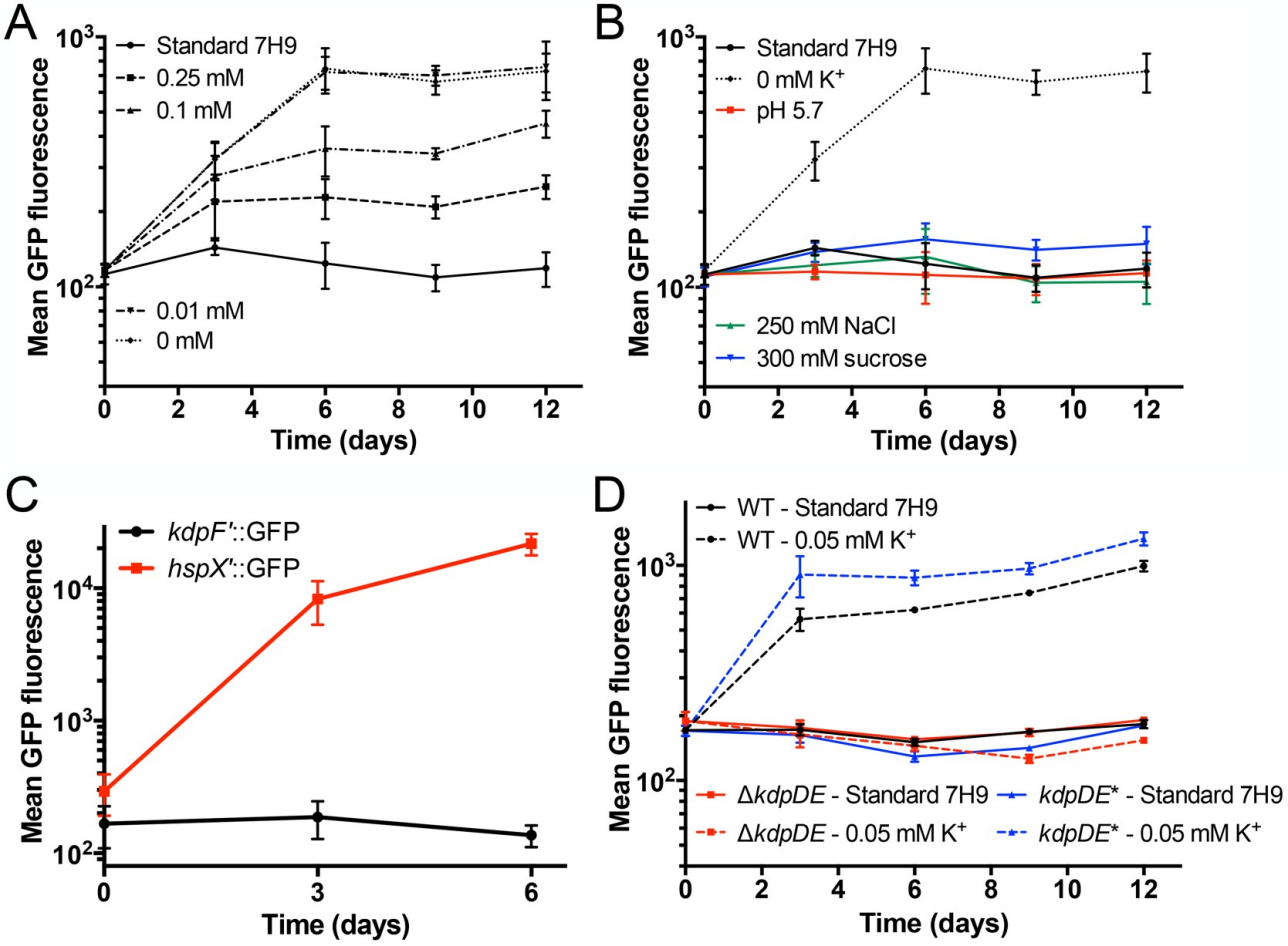
*kdpF’*::GFP functions as a specific reporter of Mtb response to environmental K^+^ levels. (A) Mtb response to K^+^ is concentration-dependent. CDC1551(*kdpF’*::GFP, *smyc’*::mCherry) was grown in standard 7H9 broth, pH 7.0, or in K^+^-free 7H9 broth, pH 7.0, supplemented with indicated amounts of K^+^. Samples were taken at indicated time points, fixed, and GFP induction analyzed by FACS. Data are shown as means ± SD from 3 independent experiments. (B-C) *kdpF’*::GFP induction is specific. CDC1551(*kdpF’*::GFP, *smyc’*::mCherry) was grown in 7H9 broth, pH 7.0, supplemented with 250 mM NaCl (green triangles) or 300 mM sucrose (blue inverted triangles), in 7H9 broth, pH 5.7 (red squares), or in K^+^-free 7H9 broth, pH 7.0 (dotted black line, circles) (B). In (C), CDC1551(*kdpF’*::GFP, *smyc’*::mCherry) was grown in media in stirred, aerated, cultures, before exposure to 0.5% O_2_ for 6 days. The hypoxia responsive strain, CDC1551(*hspX’*::GFP, *smyc’*::mCherry), was used as a control. All samples were analyzed as in (A), and data are shown as means ± SD from 3 independent experiments. (D) KdpDE inactivation abolishes *kdpF’*::GFP induction in low [K^+^]. WT, Δ*kdpDE*, or kdpDE* (complemented mutant) Mtb expressing the *kdpF’*::GFP reporter were grown in standard 7H9 broth, pH 7.0, or in K^+^-free 7H9 broth, pH 7.0, supplemented with 0.05 mM KCl. All samples were analyzed as in (A), and data are shown as means ± SD from 3 independent experiments.

The transcriptional data above demonstrated the very robust induction of the *kdpFABC* operon upon exposure to low environmental [K^+^] [24]. To aid further study of Mtb response to K^+^, we thus generated a fluorescent K^+^-responsive Mtb strain by cloning the promoter region of *kdpF* immediately upstream of GFP in a replicating plasmid, and transforming this *kdpF’*::GFP construct into Mtb. As expected, reporter GFP fluorescence was induced upon growth in low [K^+^], with signal intensity increasing with decreasing [K^+^] (Fig 2A). Testing of the *kdpF’*::GFP reporter in several other conditions known to be environmental cues for Mtb (acidic pH, high [Cl^-^], high osmolarity, and hypoxia) revealed no fluorescence induction, illustrating its specificity (Fig 2B and 2C). The lack of response to osmolarity is intriguing, as the KdpFABC system is canonically upregulated during exposure to high environmental osmolarity, in addition to limiting [K^+^] [29–31]. There is however precedence for a Kdp system being non-responsive to osmolarity, with this phenotype demonstrated for Kdp homologs in the cyanobacterium *Anabaena* and the extremophile *Deinococcus radiodurans* [32, 33]. In agreement with our results, previous studies examining the response of Mtb to osmolytes (NaCl, KCl, and sucrose) had not observed changes in *kdpFABC* gene expression [2, 34]. Mtb is extremely robust at coping with osmolarity, and it is possible that it utilizes distinct systems for osmolarity response. Indeed an osmosensory pathway regulated by the eukaryotic-like serine/threonine protein kinase PknD has been described for Mtb [34].

Expression of *kdpFABC* is expected to be modulated by the two-component system KdpDE, based on homology to model systems such as *Escherichia coli* [35], and from Mtb studies examining regulatory partners of KdpD and the effect of over-expression of *kdpE* [24, 36]. As expected by this regulation, induction of *kdpF’*::GFP signal in the presence of low environmental [K^+^] was lost in a Δ*kdpDE* Mtb mutant, with genetic complementation successfully restoring the WT phenotype (Fig 2D). These data demonstrate the robustness of the *kdpF’*::GFP reporter as a specific and sensitive K^+^ reporter for Mtb, and support its utility for analyses of Mtb K^+^ response.

### Distinct and novel shared regulatory mechanisms influence Mtb response to abundant ions

**Fig 3:**
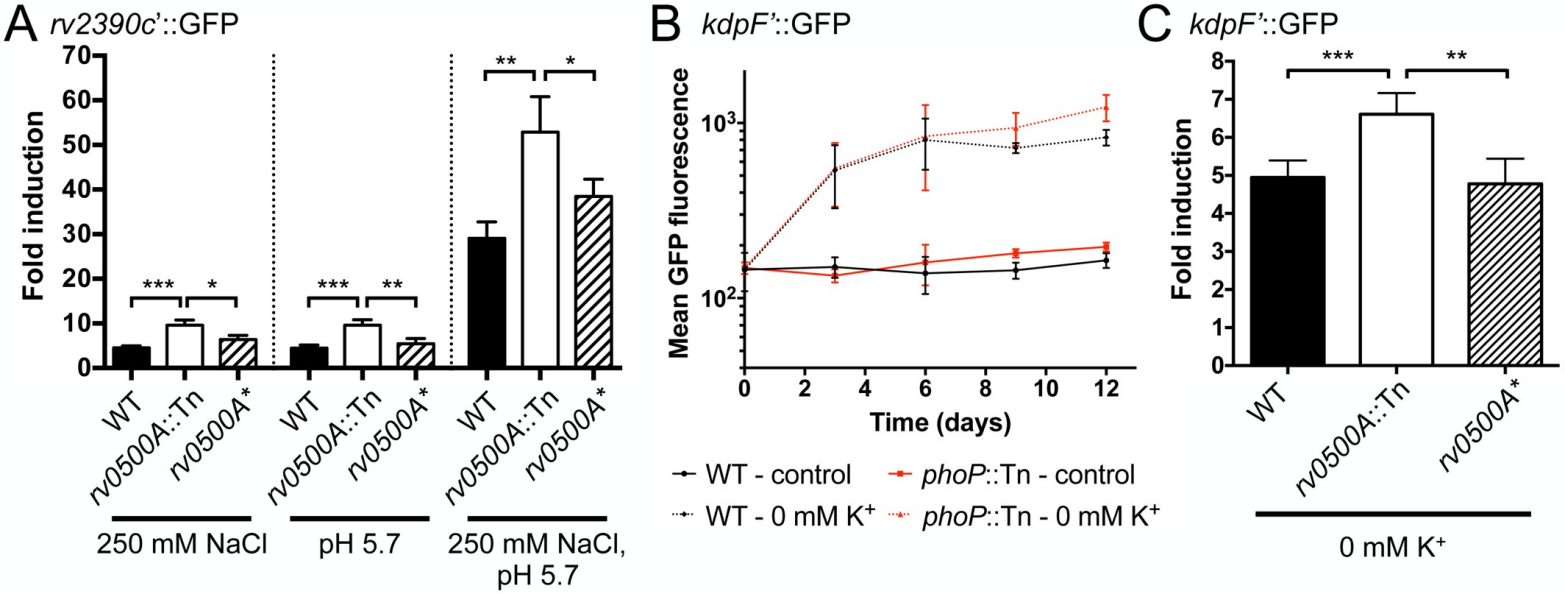
Distinct and novel shared regulatory mechanisms influence Mtb response to abundant ions. (A) *rv0500A* disruption enhances Mtb response to acidic pH and high [Cl^-^]. WT, *rv0500A*::Tn, or rv0500A* (complemented mutant) Mtb carrying the Cl^-^/pH responsive *rv2390c’*::GFP reporter were grown in pH 7 or pH 5.7 media, ± 250 mM NaCl, for 9 days. GFP signal in fixed samples was measured by FACS, with fold signal induction compared to the corresponding strain grown in pH 7 control media. Data are shown as means ± SD from 3–4 independent experiments. p-values were obtained with an unpaired t-test, * p<0.05, ** p<0.01, *** p<0.001. (B) PhoP does not affect Mtb response to K^+^. WT or *phoP*::Tn Mtb carrying the K^+^-responsive *kdpF’*::GFP reporter were grown in standard 7H9 media (control) or K^+^-free 7H9. Samples were taken at indicated time points, fixed, and GFP induction analyzed by FACS. Data are shown as means ± SD from 3 independent experiments. (C) *rv0500A* disruption enhances Mtb response to low [K^+^]. WT, *rv0500A*::Tn, or rv0500A* Mtb carrying the *kdpF’*::GFP reporter were grown in standard 7H9 media or K^+^-free 7H9 for 9 days. GFP reporter induction was determined as in (A). Data are shown as means ± SD from 3–5 independent experiments. p-values were obtained with an unpaired t-test, ** p<0.01, *** p<0.001.

With the *kdpF’*::GFP K^+^ reporter in hand, we next sought to determine if regulation of Mtb response to K^+^ is linked to the bacterial response to other abundant environmental ions, in particular H^+^ (acidic pH) and Cl^-^. Acidic pH is well-established as a critical environmental signal for Mtb during host colonization [8, 10, 37], and we had discovered that the bacteria also respond transcriptionally to external Cl^-^, with significant overlap in the pH and Cl^-^ response regulons [2]. Mtb response to pH and Cl^-^ is synergistic [2], and regulation of Mtb response to pH and Cl^-^ is closely linked, with both shared activators and repressors. In particular, we had previously found that the two-component regulator PhoPR is a key activator of Mtb response to both acidic pH and Cl^-^, with attenuation of response upon PhoPR inactivation [2]. Conversely, in an initial screen for other genes controlling Mtb response to Cl^-^ that utilized transposon libraries generated in the background of our previously described Cl^-^ and pH-sensitive *rv2390c*’::GFP reporter Mtb strain [2], we identified Rv0500A as a novel repressor of Mtb response to acidic pH and Cl^-^. Specifically, inactivation of Rv0500A was found to enhance expression of the *rv2390c’*::GFP reporter upon exposure to the inducing signals of Cl^-^ and/or acidic pH (Fig 3A).

Expression of the K^+^-sensitive *kdpF’*::GFP reporter in a *phoP* transposon mutant (*phoP*::Tn) revealed no difference in signal induction upon exposure of the bacteria to low [K^+^] as compared to WT Mtb, indicating that the PhoPR regulator does not contribute to regulation of Mtb K^+^ response (Fig 3B). In contrast, *kdpF’*::GFP expression was enhanced in a *rv0500A* transposon mutant (*rv0500A*::Tn) when the bacteria were grown in K^+^-free media (Fig 3C), similar to the enhancement of *rv2390c’*::GFP reporter signal observed during exposure of the *rv0500A*::Tn mutant to high [Cl^-^] and/or acidic pH media (Fig 3A). We note that the *rv2390c’*::GFP reporter does not respond to low external [K^+^] (S2 Fig), in accord with its previously described high specificity for Cl^-^ and pH [2].

**Fig 4:**
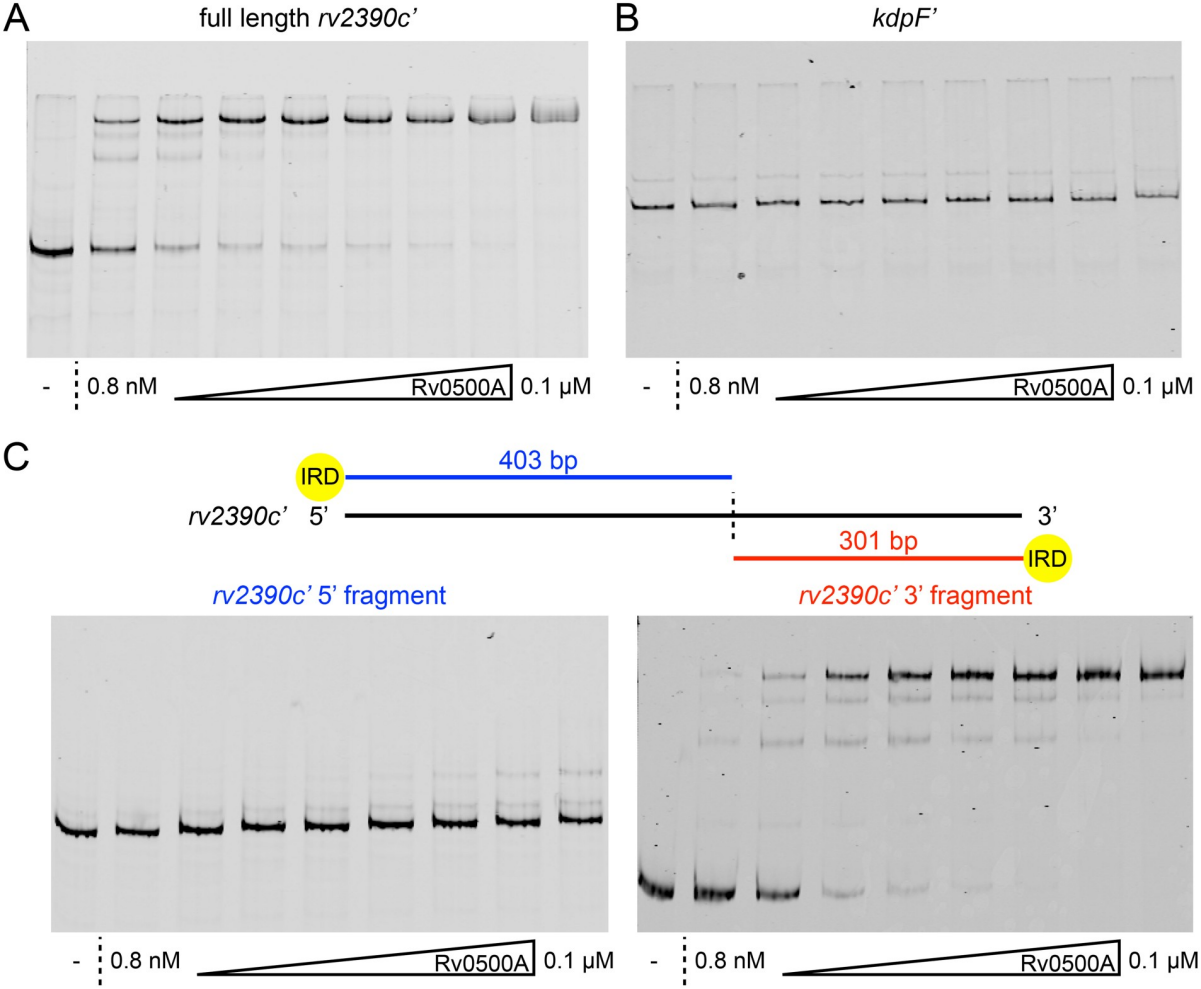
Rv0500A binds directly to the *rv2390c* promoter. (A and B) Rv0500A binds directly to the *rv2390c* promoter but not the *kdpF* promoter. Electrophoretic mobility shift assays (EMSAs) were performed using purified recombinant N-terminally 6x-His-tagged Rv0500A and DNA probes for the *rv2390c* promoter (A) or the *kdpF* promoter (B). The highest concentration binding reaction contained 0.1 μM His-Rv0500A followed by two-fold serial dilutions. A control with no His-Rv0500A protein added is shown in the first lane. All DNA probes were labeled with IRDye 700. Data are representative of 2–3 independent experiments. (C) Rv0500A binds directly to a 3’ section of the *rv2390c* promoter. The schematic at the top shows the “split” promoter regions. EMSAs were performed as above with the 5’ (left panel) or 3’ (right panel) sections of the *rv2390c* promoter. Data are representative of 2 independent experiments.

To further explore the role of Rv0500A in Mtb response to ionic signals, we expressed and purified recombinant Rv0500A (S3 Fig), and tested for its ability to directly bind the *rv2390c* and *kdpF* promoters. As shown in Fig 4A, a clear shift was observed in electrophoretic mobility shift assays (EMSAs) conducted with Rv0500A and the *rv2390c* promoter, demonstrating binding of the protein to the DNA fragment. In contrast, we did not observe a shift when the *kdpF* promoter was used (Fig 4B). EMSAs with separated 5’ and 3’ sections of the *rv2390c* promoter showed a distinct shift with the 3’ but not the 5’ section, further illustrating the specificity of Rv0500A binding (Fig 4C). Together, these data demonstrate that Rv0500A binds directly to specific DNA regions, and indicate that Rv0500A regulation of *kdpF* may be indirect, as opposed to its direct effect on *rv2390c* expression.

Our findings demonstrate that Mtb response to the different ionic signals of K^+^, Cl^-^, and pH are controlled by both distinct and shared regulatory mechanisms, and illustrate the complexity of Mtb integration of its response to disparate environmental cues. These data further reveal Rv0500A as a novel transcription regulator that represses Mtb response to these abundant ionic signals, and additional studies focused on analysis of Rv0500A function are currently in progress.

### Expression of *kdpF’*::GFP is downregulated during macrophage infection

pH and Cl^-^ are key signals present in the Mtb phagosome [2, 8, 37], and a previous study had indicated that [K^+^] in the lysosome decreases as a counterbalance to the influx of H^+^ [38]. While K^+^ levels in Mtb-containing phagosomes had been queried in an earlier intriguing study utilizing x-ray fluorescence microscopy, conclusions regarding changes in [K^+^] could not be drawn from the values obtained, and the resolution of the method had precluded differentiation of levels in the bacterium itself versus in the phagosomal compartment [39]. In the context of neutrophil phagosomes, there has been much debate as to whether [K^+^] increase is important upon generation of the oxidative burst [40–43]. Given our findings of both distinct and shared regulatory mechanisms underlying Mtb response to acidic pH, Cl^-^, and K^+^, we next utilized our *kdpF’*::GFP reporter Mtb strain as a unique tool for interrogating the relative environmental [K^+^] the bacteria are exposed to during macrophage infection.

**Fig 5:**
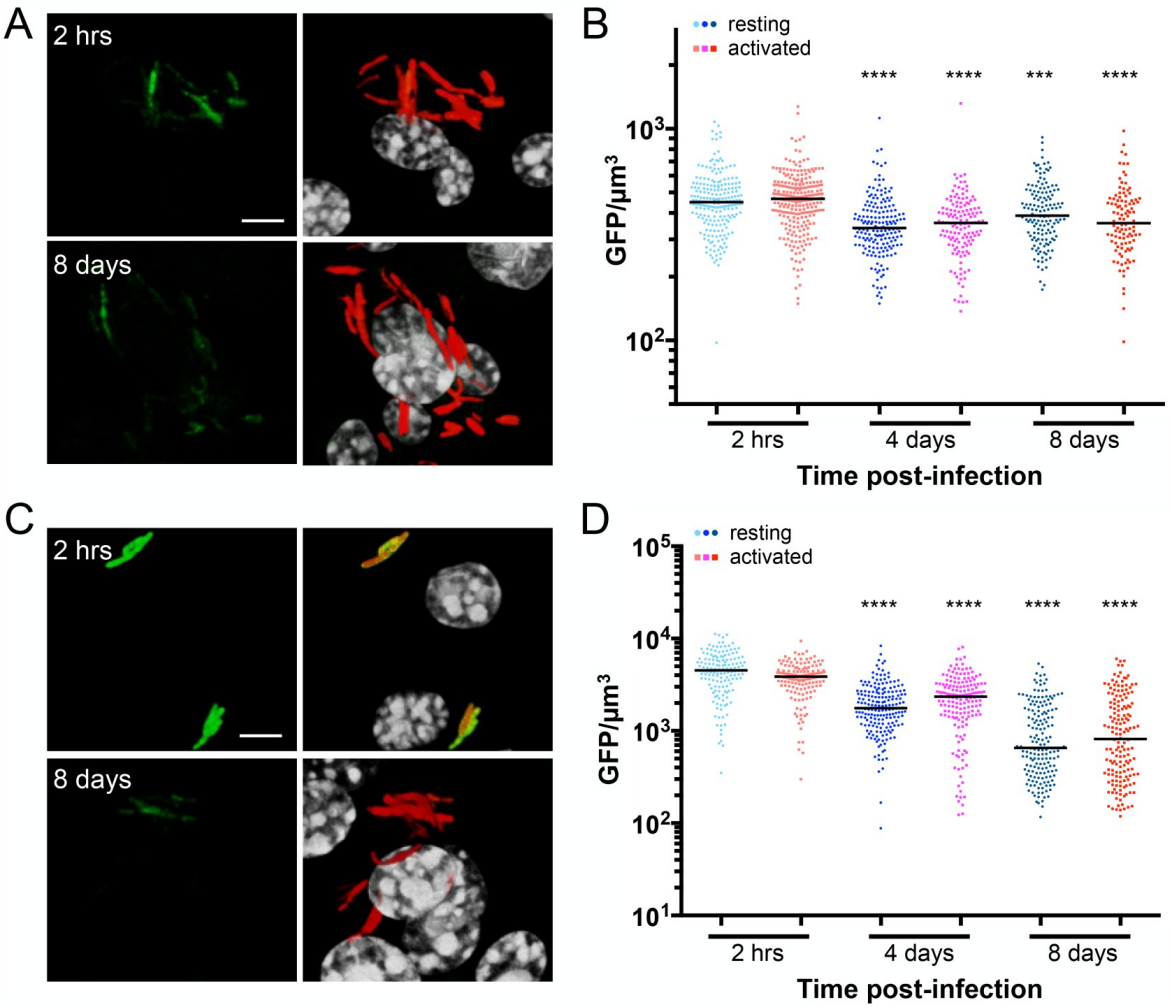
*kdpF’*::GFP is downregulated during macrophage infection. (A and B) Expression of *kdpF’*::GFP is downregulated during Mtb infection of macrophages. Resting or activated macrophages were infected with CDC1551(*kdpF’*::GFP, *smyc’*::mCherry). (A) shows 3D confocal images of the beginning (2 hrs) and end (8 days) of infection. All bacteria are marked in red (*smyc’*::mCherry), the reporter is shown in green (*kdpF’*::GFP), and nuclei are shown in grayscale (DAPI). Scale bar 5 μm. (B) shows quantification of the GFP/μm^3^ signal for each bacterium measured from multiple 3D confocal images. Each point on the graph represents a bacterium or a tight cluster of bacteria (circles—Mtb in resting macrophages, squares—Mtb in activated macrophages). p-values were obtained with a Mann-Whitney statistical test, and is in comparison to the corresponding 2 hr data set in each case. *** p<0.001, **** p<0.0001. (C and D) Pre-growth of Mtb in K^+^-free media enhances *kdpF’*::GFP reporter signal decrease during infection. CDC1551(*kdpF’*::GFP, *smyc’*::mCherry) was grown in K^+^-free media for 6 days prior to infecting resting or activated murine macrophages. (C) shows 3D confocal images of the infection at the beginning (2 hrs) and end (8 days) of the infection. All bacteria are marked in red (*smyc’*::mCherry), the reporter is shown in green (*kdpF’*::GFP), and nuclei are shown in grayscale (DAPI). Scale bar 5 μm. (D) shows quantification of the bacterial GFP/μm^3^ signal, determined as in (B).

For this assay, we used our previously established dual fluorescent reporter strategy, in which a constitutively expressed *smyc’*::mCherry is placed downstream of the environmental-responsive reporter (here *kdpF’*::GFP), enabling visualization of all bacteria during infection [2, 37, 44]. Infection of primary murine bone marrow-derived macrophages with this dual *kdpF’*::GFP, *smyc’*::mCherry reporter Mtb strain revealed a decrease in *kdpF’*::GFP reporter signal over the course of eight days in both resting and activated macrophages (Fig 5A and 5B). This downregulation in *kdpF’*::GFP signal was further emphasized when the reporter Mtb were grown in low environmental [K^+^] prior to infection (Fig 5C and 5D).

These data strongly indicate that K^+^ is not limiting within the Mtb phagosome, similar to conclusions reached for the *Salmonella*-containing vacuole that were based on transcriptional studies showing no upregulation of bacterial K^+^ transport systems in intracellular *Salmonella* [45]. Our findings further suggest that the outward K^+^ flux countering acidification of lysosomes previously observed does not function in the context of the Mtb phagosome [38], in accord with Mtb prevention of complete acidification of its phagosome [46, 47].

### Intraphagosomal K^+^ concentration increases during phagosomal maturation

**Fig 6:**
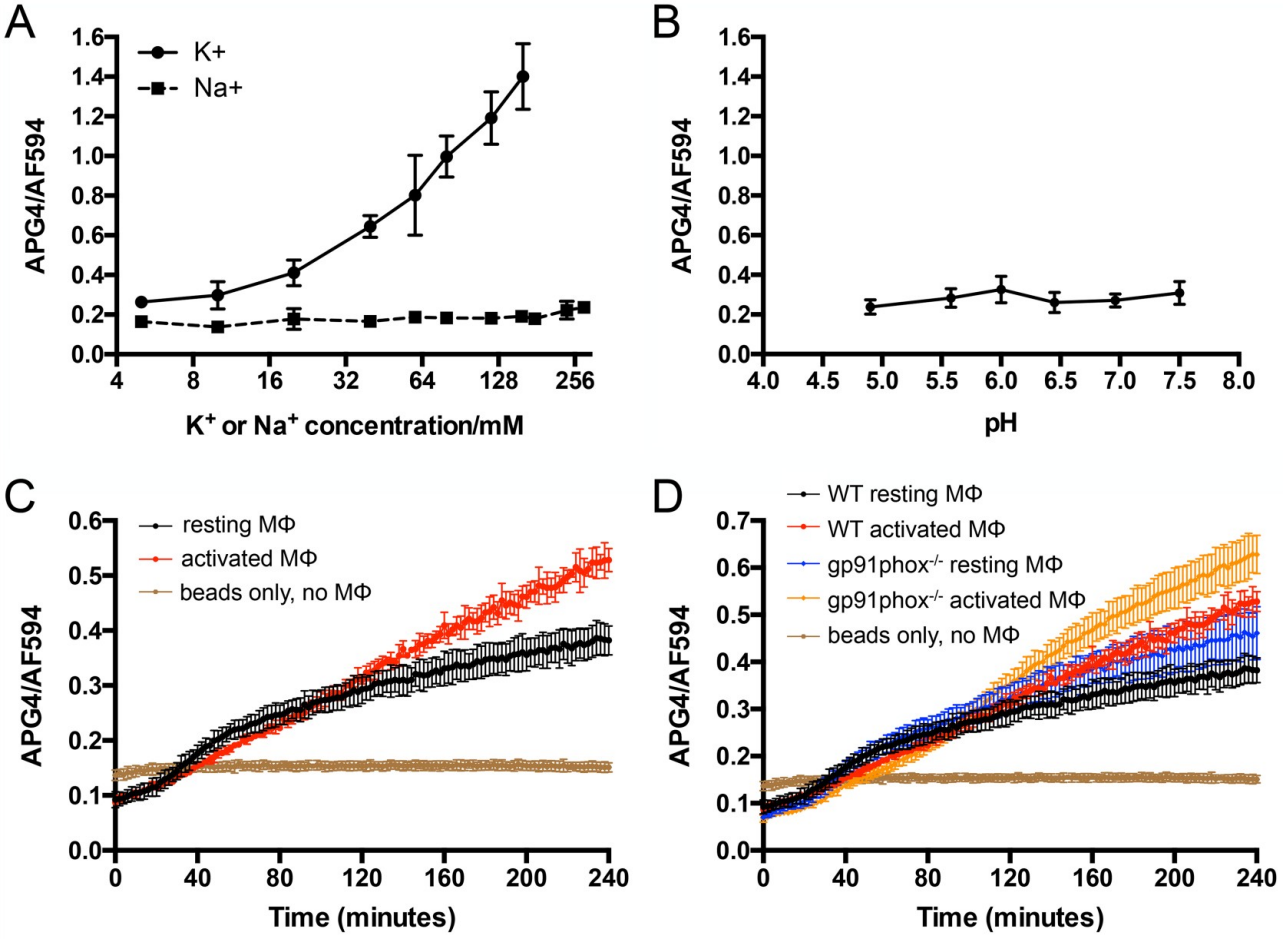
Intraphagosomal K^+^ concentration increases during phagosomal maturation. (A and B) APG4/AF594 beads respond specifically to [K^+^]. APG4/AF594 beads were placed in buffer containing specific [K^+^] or [Na^+^] (A), or in buffer with 100 mM Bis-Tris adjusted to the indicated pH with HCl (B). APG4 and AF594 fluorescence were measured on a microplate reader, and data are shown as means ± SD from 4 wells, representative of 2–3 independent experiments. (C and D) Intraphagosomal [K^+^] increases during phagosomal maturation independent of an oxidative burst. APG4/AF594 beads were added to resting or activated murine bone marrow-derived macrophages (MØ) from WT (C and D) or gp91phox^-/-^ mice (D), and fluorescence tracked with a microplate reader over time. Sensor beads were also added to wells containing only media, with no macrophages (“beads only, no MØs”). Data are shown as means ± SD from 4 wells, representative of 3–5 independent experiments.

To further examine [K^+^] changes in the maturing phagosome with an independent approach, we utilized the K^+^-sensitive compound Asante K^+^ Green 4 (APG4) [22, 48], which we covalently linked to 3 μm silica beads, together with Alexa Fluor 594 (AF594) as a calibration fluorophore [2]. These K^+^ sensor beads fluoresced specifically as [K^+^] increased, with no APG4 fluorescence induced by changes in sodium concentration or pH (Fig 6A and 6B). In agreement with the *kdpF’*::GFP reporter assay above, tracking of APG4/AF594 bead fluorescence upon addition to primary murine bone marrow-derived macrophages showed that K^+^ was not limiting in the maturing macrophage phagosome; instead, phagosomal [K^+^] increased as the compartment matured in both resting and activated macrophages (Fig 6C). Inhibition of H^+^-ATPase activity with concanamycin A inhibits phagosome acidification and had previously been shown to affect phagosome maturation [49]. We found that concanamycin A treatment resulted in very significant loss of the increase observed in APG4/AF594 signal in mock-treated samples, further supporting the conclusion that [K^+^] increases during phagosome maturation (S4 Fig).

As the oxidative burst has been implicated as a driving factor in [K^+^] increase within neutrophil phagosomes [40], we next asked if macrophages deficient in NADPH oxidase function (gp91phox^-/-^) would exhibit the increase in phagosomal [K^+^] observed in WT macrophages. We found that gp91phox^-/-^ macrophages retained their ability to increase phagosomal [K^+^], indicating that the oxidative burst is not required for this phenotype in macrophages (Fig 6D). These findings add to the knowledge base of changes in the ionic milieu during macrophage phagosome maturation, and further studies are required to delineate the channels/transporters that mediate this K^+^ flux.

### Mtb K^+^ homeostasis augments the bacterial response to Cl^-^ and pH

Having established that [K^+^] increases during macrophage phagosome maturation, similar to increases in [Cl^-^] and [H^+^] [2], and in light of the interplay in Mtb response to these ions illustrated by our results above with Rv0500A, we next sought to determine if disruption of Mtb K^+^ homeostasis would affect bacterial response to Cl^-^ and pH. Mtb has two K^+^ uptake systems: (i) Kdp, a high affinity, inducible system, and (ii) Trk, a constitutive, low-moderate affinity system [23, 50, 51]. Given our finding that K^+^ is not limiting within the Mtb phagosome, we focused our studies here on the Trk K^+^ uptake system, encoded by the *ceoB* and *ceoC* genes.

**Fig 7:**
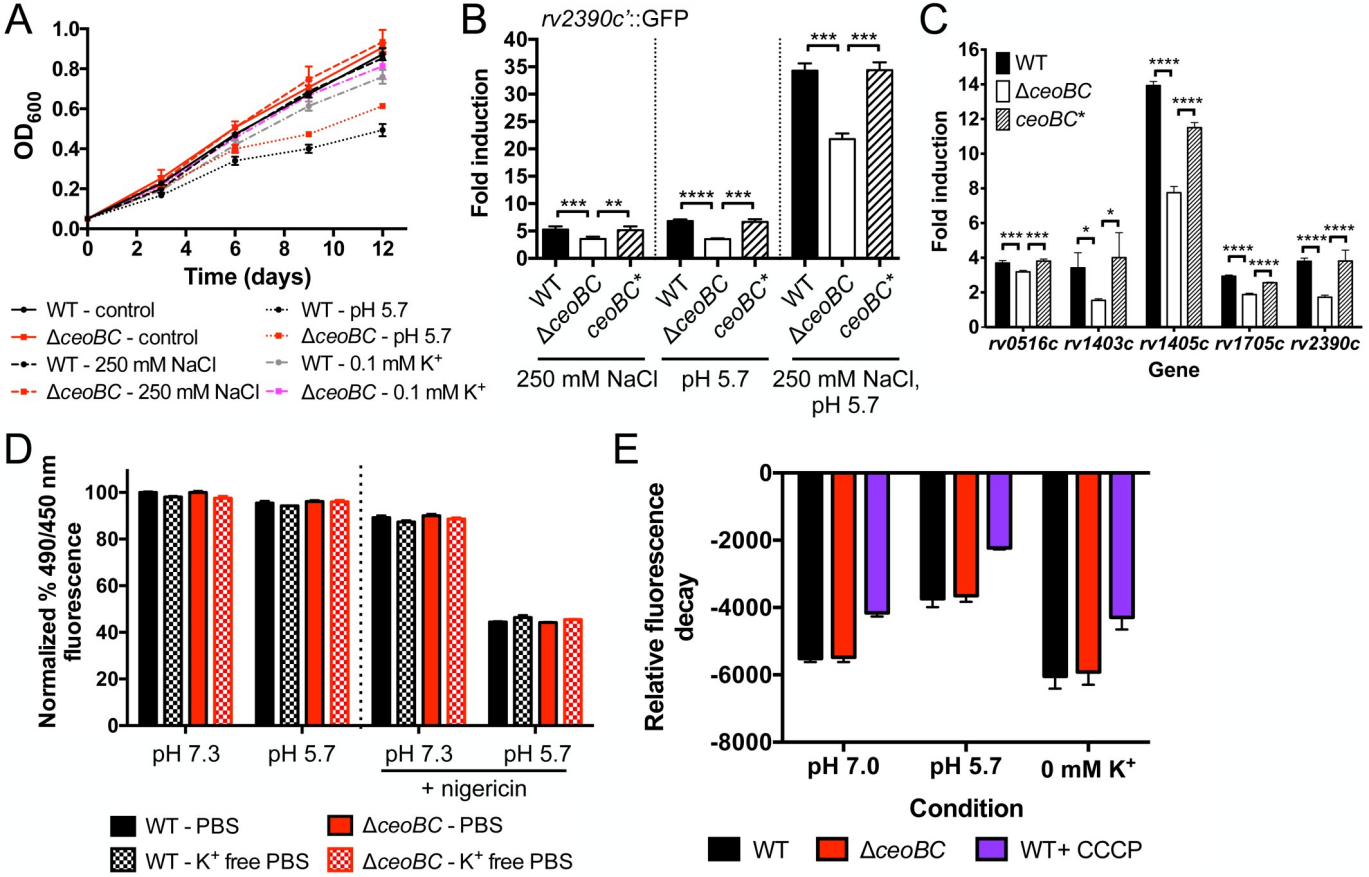
Mtb K^+^ homeostasis augments the bacterial response to Cl^-^ and pH. (A) Mtb Δ*ceoBC* mutant growth during environmental stress. WT or Δ*ceoBC* mutant Mtb were grown in standard pH 7.0 media ± 250 mM NaCl, pH 5.7 media, or in K^+^-free 7H9 media, pH 7.0, supplemented with 0.1 mM KCl, and growth tracked over time. Data are shown as mean ± SD from 3 independent experiments. (B and C) Mtb Δ*ceoBC* mutant has an attenuated response to Cl^-^ and acidic pH. In (B), WT, Δ*ceoBC*, or ceoBC* (complemented mutant) Mtb expressing the *rv2390c’*::GFP reporter were grown in pH 7 or pH 5.7 media, ± 250 mM NaCl. GFP signal in fixed samples was measured by FACS, with fold signal induction compared to the corresponding strain grown in pH 7 control media. Data are shown as means ± SD from 3 independent experiments. p-values were obtained with an unpaired t-test, ** p<0.01, *** p<0.001, **** p<0.0001. (C) shows qRT-PCR of gene expression changes in WT, Δ*ceoBC*, or ceoBC* 4 hrs post-exposure to pH 7 media ± 250 mM NaCl. Fold induction is as compared to gene expression in pH 7 media for each strain. Data are shown as mean ± SD from 3 technical replicates, representative of 2 independent experiments. p-values were obtained with an unpaired t-test, * p<0.05, *** p<0.001, **** p<0.0001. (D) Maintenance of intrabacterial pH in the Δ*ceoBC* mutant. WT or Δ*ceoBC* mutant Mtb were incubated for 1 hour with 5-chloromethylfluorescein diacetate, before washing and exposure to PBS or K^+^-free PBS containing 0.05% tween 80, at pH 7.3 or pH 5.7. Fluorescence was read with a microplate reader at Ex. 450 nm and 490 nm, and Em. at 520 nm. 10 μM of the ionophore nigericin was added to assay control samples. Data were normalized as a percentage of the fluorescence ratios (Ex. 490 nm/Ex, 450 nm) observed in PBS, pH 7.3, for a given strain, and shown as means ± SD from 3 wells. The results are representative of 4 independent experiments. (E) Δ*ceoBC* mutant maintains membrane potential during environmental stress. WT or Δ*ceoBC* mutant Mtb were resuspended in pH 7 or pH 5.7 media, or in K^+^-free 7H9, pH 7 media containing 0.5 mg/l of rhodamine 123. 50 μM of the protonophore carbonyl cyanide *m*-chlorophenylhydrazone was added to assay control samples. Fluorescence decay was tracked every 10 minutes for 4 hours with a microplate reader at Ex. 485 nm/Em. 527 nm, and normalized to initial probe fluorescence for each strain and condition. The assay was repeated 4 times, and data are shown as means ± SD from 8 wells across 2 independent experiments.

A Δ*ceoBC* Mtb mutant was constructed via homologous recombination, and the Cl^-^ and pH-responsive *rv2390c’*::GFP reporter introduced into the strain [2]. In agreement with the role of CeoBC as a constitutive, low-moderate affinity K^+^ uptake system, tests with the *kdpF’*::GFP reporter indicated that the Δ*ceoBC* mutant is slightly more sensitive to low environmental [K^+^] (S5 Fig). The Δ*ceoBC* mutant had no growth defect in standard broth, or in high [Cl^-^], acidic pH, or low [K^+^] media, as compared to WT Mtb (Fig 7A). Strikingly however, the Δ*ceoBC* mutant demonstrated significantly decreased reporter GFP signal when grown in defined broth conditions with high [Cl^-^] and/or acidic pH, which was restored to WT levels upon genetic complementation (ceoBC*) (Fig 7B). To establish that an attenuated response to environmental [Cl^-^] and pH by the Δ*ceoBC* mutant was a broader phenotype not restricted to *rv2390c* response, we performed qRT-PCR on several other Cl^-^ response regulon genes [2]. As shown in Fig 7C, the transcriptional response of the Δ*ceoBC* mutant was dampened in each case, although the magnitude of reduction varied from gene to gene.

To test that the phenotypes observed were due more directly to disruption of bacterial K^+^ homeostasis versus possible downstream effects on the bacterial membrane from deletion of an ion transport system, we first assessed the ability of the Δ*ceoBC* mutant to maintain intracellular pH during growth in acidic conditions. As previously described, the pH-sensitive, cell-permeable, dye 5-chloromethylfluorescein diacetate (CMFDA) is rendered cell-impermeant once in the cytoplasm of cells, and thus enables measurement of intracellular pH in a ratiometric manner, as fluorescein is pH sensitive when excited at 490 nm, but pH insensitive when excited at 450 nm (emission at 520 nm in both cases) [52]. We found that the Δ*ceoBC* mutant maintained intracellular pH as well as WT Mtb, even when exposed to a pH 5.7 environment (Fig 7D). Treatment of the bacteria with nigericin, an ionophore, resulted in the expected decrease in excitation 490 nm/450 nm (emission 520 nm) fluorescence ratio in the presence of external acidity, and served as an assay control (Fig 7D).

Next, we tested for defects in maintenance of membrane potential. The cationic dye Rhodamine 123 allows relative determination of cellular membrane potential, with increased uptake, and consequently greater fluorescence decay over time, in cells with higher membrane potential [53, 54]. In standard pH 7 growth media, WT and Δ*ceoBC* Mtb had similar membrane potential (Fig 7E). Exposure of Mtb to an acidic external pH resulted in the expected membrane depolarization, but here again no difference in membrane potential was observed between WT and Δ*ceoBC* Mtb (Fig 7E). Membrane potential of the Δ*ceoBC* mutant was also not different to that of WT Mtb in conditions of low environmental [K^+^] (Fig 7E). The effect of protonophore (carbonyl cyanide *m*-chlorophenylhydrazone, CCCP) addition was analyzed as a control, and resulted in the expected membrane depolarization and decrease in relative fluorescence decay in each condition (Fig 7E).

These results reveal the novel concept that bacterial K^+^ homeostasis can impact on Mtb response to ionic signals in its local environment, independent of broader effects on maintenance of membrane potential. This new concept highlights the complexity of bacterial K^+^ biology, and the intimate relationship between ionic homeostasis and ionic signal response. It further raises the intriguing suggestion that targeting of Mtb K^+^ homeostasis will have far-reaching adverse consequences for the bacterium that extend beyond K^+^ balance alone.

### Disruption of K^+^ homeostasis attenuates Mtb colonization ability in macrophages and *in vivo*

**Fig 8:**
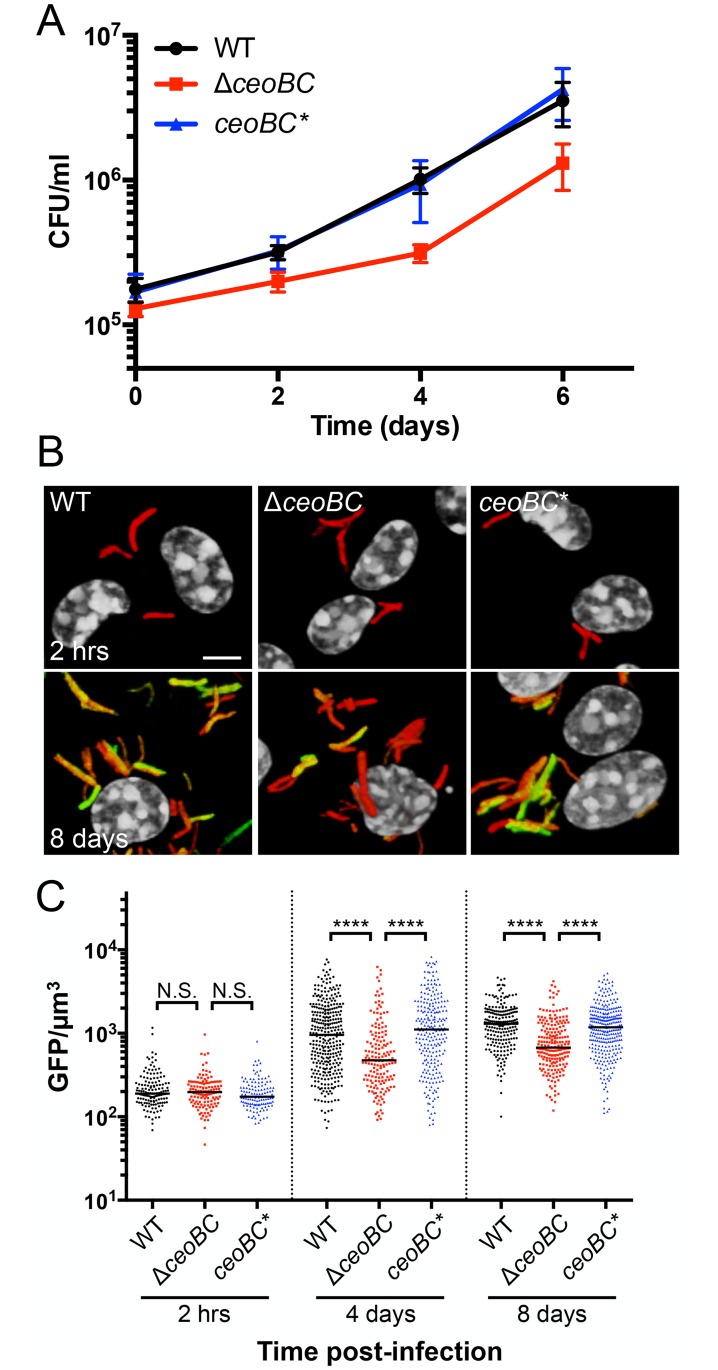
Disruption of bacterial K^+^ homeostasis decreases Mtb colonization of macrophages. (A) Mtb Δ*ceoBC* mutant is attenuated for macrophage colonization. Murine bone marrow-derived macrophages were infected with WT, Δ*ceoBC*, or ceoBC* (complemented mutant) Mtb, and CFUs tracked over time. Data are shown as means ± SD from 5–6 wells, representative of 3–4 independent experiments. (B and C) Mtb Δ*ceoBC* mutant exhibits decreased *rv2390c’*::GFP induction response during macrophage infection. Activated murine bone marrow-derived macrophages were infected with WT, Δ*ceoBC*, or ceoBC* Mtb expressing the *rv2390c’*::GFP, *smyc’*::mCherry reporter. (B) shows 3D confocal images of the beginning (2 hrs) and end (8 days) of infection. All bacteria are marked in red (*smyc’*::mCherry), the reporter is shown in green (*rv2390c’*::GFP), and nuclei are shown in grayscale (DAPI). Scale bar 5 μm. (C) shows quantification of the GFP/μm^3^ signal for each bacterium measured from multiple 3D confocal images. Each point on the graph represents a bacterium or a tight cluster of bacteria (black circles—WT, red squares– Δ*ceoBC*, blue triangles—ceoBC*). p-values were obtained with a Mann-Whitney statistical test. N.S. not significant, **** p<0.0001.

The changing [K^+^] in the phagosome and the observed impairment in bacterial response to pH and Cl^-^ in the Δ*ceoBC* mutant suggests that this mutant may be attenuated in colonization of macrophages. To test this, we infected primary murine bone marrow-derived macrophages with WT, Δ*ceoBC*, or ceoBC* Mtb, and tracked bacterial growth over time. We found that the Δ*ceoBC* mutant was significantly attenuated in its ability to colonize macrophages, with the bacterial load recovered 6 days post-infection only about half that obtained from WT or ceoBC*-infected samples (Fig 8A). In agreement with our results, we note that *ceoB* mutants had previously been found to be under-represented in a transposon site hybridization-based screen for Mtb genes important for survival/growth in macrophages [55]. Utilization of the *rv2390c’*::GFP, *smyc’*::mCherry reporter in the various strains further showed that *rv2390c’*::GFP signal was decreased in the Δ*ceoBC* mutant as compared to that in WT or ceoBC* Mtb during infection of activated macrophages (Fig 8B and 8C). These reporter data suggest that the impaired response of the Δ*ceoBC* mutant to Cl^-^ and pH observed in broth is similarly exhibited during macrophage infection.

**Fig 9:**
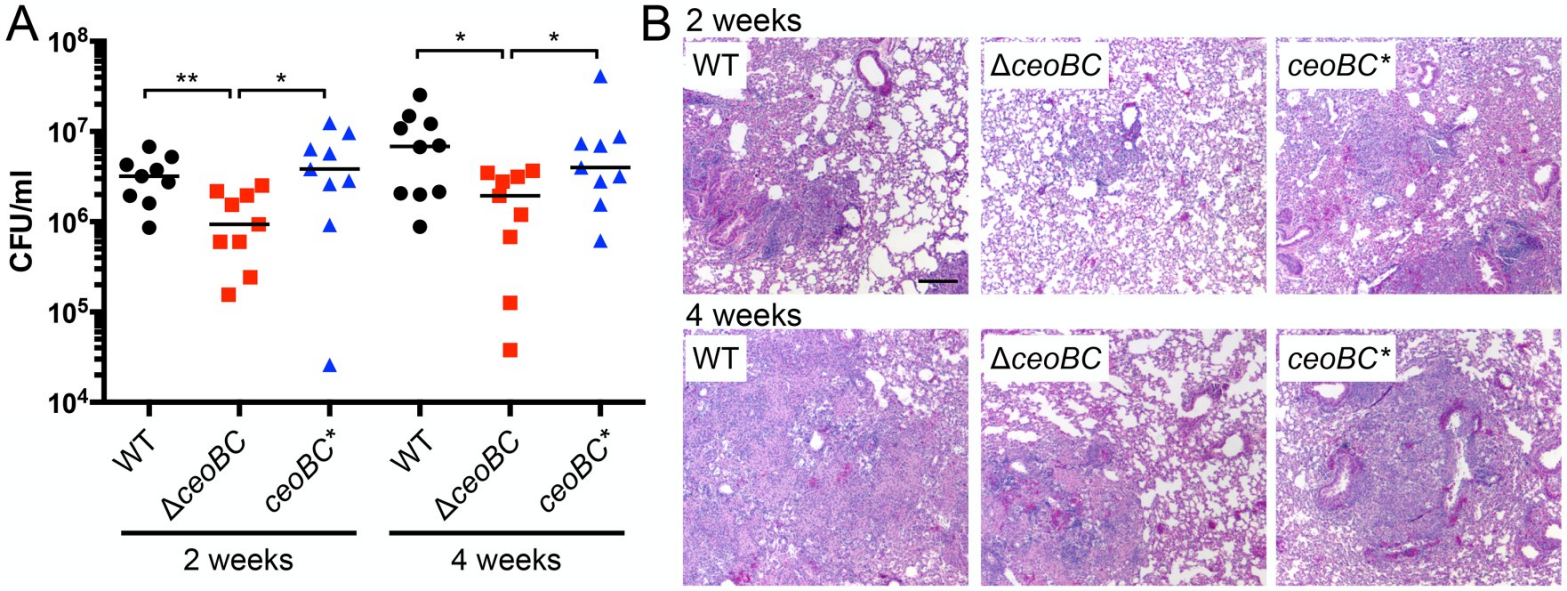
Disruption of bacterial K^+^ homeostasis results in attenuation of Mtb growth *in vivo*. (A) Mtb Δ*ceoBC* mutant is attenuated for colonization in a murine infection model. C57BL/6J WT mice were infected with WT, Δ*ceoBC*, or ceoBC* (complemented mutant) Mtb, and lung homogenates plated for CFUs 14 or 28 days post-infection. p-values were obtained with a Mann-Whitney statistical test. * p<0.05, ** p<0.01. (B) Lung pathology in infected mice. Lungs obtained from infected animals 14 or 28 days post-infection were fixed and processed for hematoxylin and eosin staining. Scale bar 200 μm.

Finally, to examine the effect of disrupting Mtb K^+^ homeostasis on the bacterium’s ability to colonize a whole animal host, we infected C57BL/6J WT mice with WT, Δ*ceoBC*, or ceoBC* Mtb, and analyzed bacterial load and histopathology two and four weeks post-infection. The Δ*ceoBC* mutant was significantly attenuated for host colonization at both time points, with bacterial loads 3–4 fold less than that obtained from infections with WT or ceoBC* (Fig 9A). Histopathology in Δ*ceoBC*-infected animals was correspondingly less severe, with decreased cellular infiltration and better maintenance of clear alveolar air spaces (Fig 9B). These results demonstrate a role for the CeoBC K^+^ uptake system in Mtb pathogenesis, and underscore the importance of K^+^ homeostasis for the bacterium in successfully establishing host colonization.

### Concluding remarks

From the macrophage phagosome to the granuloma, Mtb is exposed to a complex environmental milieu that plays a significant role in its infection biology. Signals present reflect cellular and tissue location, as well as the host immune status [1–4], and Mtb must coordinate its response to disparate signals to ensure adaptation and continued survival and growth. Our discovery that disruption of the CeoBC K^+^ uptake system results in attenuation of Mtb response to external pH and Cl^-^ levels, independent of changes in membrane potential, reveals the close links between ionic homeostasis and bacterial response to diverse environmental cues. Together with our previous findings demonstrating the synergistic response of Mtb to acidic pH and Cl^-^ [2], these results highlight the need to examine Mtb ionic homeostasis and response beyond the study of individual signals in isolation. Our discovery of Rv0500A as a shared repressor affecting Mtb response to pH, Cl^-^, and K^+^ begins to shed mechanistic insight into this key facet, and studies to elucidate the role of Rv0500A during infection are ongoing. Interestingly, a global transcription factor study by Rustad *et al*. identified over-expression of *glnR* (*rv0818*), a master nitrogen metabolism regulator [56, 57], as inducing transcription of *kdpFABC* in standard broth conditions where this K^+^ uptake system is not normally upregulated [36]. It will thus also be intriguing in future studies to assess the connections that may exist between K^+^ signals and nitrogen metabolism proposed by this data. In addition, the observed attenuation of the Δ*ceoBC* mutant in host colonization suggests that uncovering how Mtb integrates ionic homeostasis and response to environmental cues represents an important area for understanding mechanisms that enable effective bacterial colonization of the host, essential for complete comprehension of Mtb pathogenesis. Our findings are likely also applicable to other bacterial pathogens, given the widespread presence of the Trk (CeoBC) K^+^ uptake system, which in the case of *Salmonella* has also been shown to play an important role during infection [20].

More broadly, studies on the role of ions in host-pathogen interactions have most often focused on scarce ions that can be sequestered by the host, such as iron, zinc, and manganese [6, 7, 58, 59]. These ions are clear targets in understanding bacterial pathogenesis, given both their scarcity and their importance for bacterial and host physiology. Much less studied however has been the role that abundant ions, like K^+^ and Cl^-^, can play in infectious disease. While such ions are not a resource that bacteria have to “fight” their host for, we propose that their very abundance, inability to be sequestered by the host, varying levels in different cellular compartments and upon immune activation, and critical roles in host biology, make them ideal targets for exploitation by pathogens. Our findings of the change in [K^+^] during macrophage phagosome maturation, and of the specific transcriptional response of Mtb to K^+^, builds on our previous results with Cl^-^ [2], and raise questions about how bacteria may exploit [K^+^] flux during infection. *S*. *enterica* presents a noteworthy example, given the reported increase in virulence factor expression upon exposure to higher environmental [K^+^] [19]. Might external K^+^ levels also affect the ability of Mtb to adapt to its local environment? It further remains to be elucidated when Mtb may experience limiting environmental [K^+^], and the possible link to iron and oxidative stress suggested by our transcriptional data is an intriguing finding that merits further study. Of note, K^+^ levels have been implicated in macrophage immune responses, with inhibition of K^+^ channels reducing nitric oxide induction [60], and low intracellular K^+^ activating the NALP3 inflammasome [61]. K^+^ may thus function as a signal both for the host and the bacterium, and further studies are needed to determine crosstalk and balance in these processes during host-pathogen interactions.

We propose that further studies pursuing the novel concepts highlighted by our results here will provide important insight into how fundamental aspects of both Mtb and host biology are linked during the infection process, and reveal unique nodes that can be perturbed to shift the balance of infection.

## Materials and methods

### Ethics statement

All animal procedures followed the National Institutes of Health “Guide for the Care and Use of Laboratory Animals” standards. The Institutional Animal Care and Use Committee at Tufts University reviewed and approved all animal protocols (#B2016-37), in accordance with guidelines established by the Association for Assessment and Accreditation of Laboratory Animal Care, the US Department of Agriculture, and the US Public Health Service.

### Mtb strains and culture

Mtb cultures were propagated as previously described [37]. Strains for *in vitro* assays were in the CDC1551 background, while strains for CFU enumeration in macrophages and *in vivo* were in the Erdman background. Mtb mutants and their complements were constructed as previously described [2]. The Δ*ceoBC* mutation consisted of a deletion beginning 102 bp from the *ceoB* start codon through 73 bp from the *ceoC* stop codon, while the entire open reading frame was deleted for Δ*kdpDE*. The *phoP*::Tn mutant from BEI Resources (NR-14776) has been previously described [2, 37]. The *rv0500A*::Tn mutant was isolated from a Tn library generated in the background of a CDC1551(*rv2390c’*::GFP) strain via use of a *mariner*-based ϕMycoMarT7 phage [62, 63], with the Tn insertion 125 bp into the open reading frame.

### RNA sequencing and qRT-PCR analyses

Log-phase (OD_600_ ~ 0.6) Mtb was used to inoculate standing, vented, T-25 flasks containing either 10 ml of standard 7H9, pH 7 medium ([K^+^] = 7.35 mM) or K^+^-free 7H9, pH 7 medium at OD_600_ = 0.3. K^+^-free 7H9 was made by replacing monopotassium phosphate with monosodium phosphate. To make Na^+^-free 7H9, disodium phosphate was replaced with dipotassium phosphate, monosodium glutamate with monopotassium glutamate, and sodium citrate with potassium citrate. The media was supplemented with 0.5% bovine serum albumin, 0.2% dextrose, and 14.5 mM KCl, and brought to pH 7 with KOH. Samples were collected 4 hours post-exposure and RNA isolation carried out as previously described [8]. Two biological replicates per condition were used for RNA sequencing. Library preparation using Ribo-Zero rRNA removal (bacterial) and TruSeq Stranded kits (Illumina) were performed by the Tufts University Genomics Core Facility. Barcoded samples were pooled and run on a single lane on an Illumina HiSeq 2500 (High Output v4) with single-end 100 bp reads. Data were analyzed using the SPARTA program [64]. qRT-PCR experiments were performed as previously described, except cDNA was synthesized from 250 ng of RNA without prior amplification [2, 37].

### Fluorescent reporter Mtb strains and *in vitro* assays

The Cl^-^ and pH-sensitive *rv2390c’*::GFP reporter has been previously described [2]. A similar approach was used to construct the K^+^-sensitive reporter *kdpF’*::GFP, with the 754 bp region immediately upstream of *kdpF* amplified by PCR and fused to GFPmut2 [2, 37]. The resulting *kdpF’*::GFP construct was then introduced into a vector containing the constitutively expressed *smyc’*::mCherry, and transformed into Mtb. Selection was carried out on 7H10 agar containing 25 μg/ml kanamycin, 50 μg/ml apramycin, or 50 μg/ml hygromycin B as appropriate.

Broth culture assays were conducted by growing Mtb in standing vented T-25 flasks in 10 ml of 7H9 buffered media as previously described [2], with addition of 250 mM NaCl or 300 mM sucrose as needed. To vary [K^+^], a specified amount of KCl was added to the K^+^-free 7H9. Antibiotics were added as necessary to maintain selection. The hypoxia assays were performed as previously described [2]. Mtb was fixed in 4% paraformaldehyde (PFA) in phosphate buffered saline (PBS) prior to fluorescence analysis. GFP was read on a BD FACSCalibur with subsequent data analysis using FloJo (Tree Star, Inc).

### Expression and purification of recombinant Rv0500A

The *rv0500A* open reading frame from CDC1551 was cloned into pET-28a (Novagen) to construct an in-frame N-terminal 6x-His tag fusion. A sequence verified construct was then transformed into *E*. *coli* BL-21 (DE3). For expression, a single transformant colony from an LB plate containing 50 μg/ml kanamycin was picked and grown overnight at 37°C on a roller drum in 2.5 ml of LB broth + 50 μg/ml kanamycin. 1 L of 2YT (5 g NaCl, 10 g yeast extract, and 15 g tryptone per liter) with 50 μg/ml kanamycin was subsequently inoculated with 2 ml of the overnight culture and grown shaking at 37°C to an OD_600_ of 0.75. To induce protein expression, 1 mM isopropyl-β-D-1-thiogalactopyranoside (IPTG) was added, and the culture grown overnight, shaking, at 18°C. Induced culture was pelleted the next day and resuspended in 25 ml lysis buffer (500 mM NaCl, 50 mM Tris pH 7.5, 15 mM imidazole, 10% glycerol). The resuspended cells were flash frozen in liquid nitrogen, thawed, and lysed via sonication. The insoluble fraction was pelleted and the cleared lysate mixed with 1 ml of Ni-NTA agarose (Macherey-Nagel) and incubated overnight at 4°C on a nutator. Purified protein was eluted the next day using high-imidazole buffer (500 mM NaCl, 50 mM Tris pH 7.5, 200 mM imidazole, 10% glycerol) (elution repeated 8 times in total). Samples were run on a 20% SDS-PAGE gel, visualized with Coomassie Brilliant Blue R-250 (Bio-Rad) staining, and imaged using the 700 nm channel of an Odyssey CLx imaging system (LI-COR). Protein concentration was quantified using a Bradford assay (Bio-Rad).

### Electrophoretic mobility shift assays

To assess DNA binding of Rv0500A, promoter regions for *rv2390c* (full length—704 bp; 5’ section—403 bp; 3’ section—301 bp) and *kdpF* (754 bp) were amplified using IRDye 700 labeled primers (Integrated DNA Technologies) and the PCR products purified using a QIAquick PCR purification kit (Qiagen). Indicated amounts of purified His-Rv0500A were mixed with no more than 40 fmoles of DNA in EMSA buffer (20 mM Tris-HCl, pH 8, 50 mM KCl, 2 mM MgCl_2_, 5% glycerol, 0.5 mM EDTA, 1 mM DTT, 0.05% Nonidet P-40, 25 μg/ml salmon sperm DNA [65]) in 11 μl final volume reactions. After incubation at room temperature for 20 minutes, the reactions were run on a non-denaturing 8% Tris-glycine gel in HEPES-imidazole buffer (35 mM HEPES, 43 mM imidazole). The gel was then imaged using the 700 nm channel of an Odyssey CLx imaging system (LI-COR).

### Intrabacterial pH assay

Mtb grown to mid-log phase in standard 7H9 broth, pH 7, was pelleted and resuspended to OD_600_ = 0.6 in 10 ml of PBS (1.54 mM KH_2_PO_4_, 2.71 mM Na_2_HPO_4_, 155 mM NaCl, pH 7.3) containing 0.05% Tween 80 and 10 μM 5′-chloromethylfluorescein diacetate (Invitrogen) for 1 hour, protected from light. Bacteria were washed twice in their respective buffer type, before resuspension to OD_600_ = 1.2 in 500 μl of appropriate buffer (PBS, pH 7.3 or pH 5.7; K^+^-free PBS, pH 7.3 or pH 5.7). To generate K^+^-free PBS, NaH_2_PO_4_ was substituted for KH_2_PO_4_. For control samples, 10 μM of the ionophore nigericin was added. 100 μl samples were transferred to a 96-well clear bottom black plate (Corning Costar), with each condition read in triplicate. After equilibration, top read fluorescence at Ex. 450/10 nm, Em. 520/10 nm, and Ex. 490/10 nm, Em. 520/10 nm, was measured using a Biotek Synergy Neo2 microplate reader. The ratio of fluorescence signal at Ex. 490 nm, Em. 520 nm, versus that at Ex. 450 nm, Em. 520 nm, was used to determine relative intrabacterial pH. Data were normalized as a percentage of the fluorescence ratios observed in PBS, pH 7.3, for a given strain [52].

### Bacterial membrane potential assay

Mtb grown to mid-log phase were resuspended to OD_600_ = 0.6 in (i) 7H9, pH 7, (ii) 7H9, pH 5.7, or (iii) K^+^-free 7H9, pH 7, with each containing 0.5 mg/l rhodamine 123 (Sigma) [53]. 50 μM carbonyl cyanide *m*-chlorophenylhydrazone (Sigma) was added for control samples. 150 μl quadruplicate aliquots of the bacterial suspensions were then transferred to a 96-well clear bottom black plate. Fluorescence decay was tracked every 10 minutes for 4 hours with a Biotek Synergy Neo2 microplate reader (bottom read Ex. 485/10 nm, Em. 527/10 nm), and normalized to initial probe fluorescence for each strain and condition.

### Macrophage culture and infections

C57BL/6J wild type and gp91phox^-/-^ mice (Jackson Laboratories) were used for isolation of bone marrow-derived macrophages. Cells were maintained in DMEM supplemented with 10% FBS, 15% L-cell conditioned media, 2 mM L-glutamine, 1 mM sodium pyruvate, and penicillin/streptomycin as needed, in a 37°C incubator at 5% CO_2_. Macrophage infections with Mtb were performed as previously described [2, 37]. For CFU enumeration, macrophages were lysed with water containing 0.01% sodium dodecyl sulfate. The strain expressing *kdpF’*::GFP was pre-induced for 6 days in K^+^-free 7H9, pH 7, prior to infection as needed.

### Confocal immunofluorescence microscopy

Fixed Mtb-infected macrophages were stained with DAPI and Alexa Fluor 647 phalloidin (Invitrogen) for visualization of nuclei and F-actin respectively. Stained samples were mounted with ProLong Diamond antifade, and images acquired with Leica LAS X software using a Leica SP8 confocal laser scanning microscope, with 0.5 μm z-steps. 3D reconstruction and image analyses were carried out with Volocity software (PerkinElmer) as previously described [2, 66, 67].

### Generation of K^+^ beads

K^+^ sensor beads were generated by first covalently linking human IgG (Sigma) and fatty acid-free bovine serum albumin (Sigma) to 12.5 mg of carboxylated, 3 μm silica beads (Kisker Biotech) as previously described [2, 68]. Beads were then washed once with coupling buffer (0.1 M sodium borate, pH 8.0), and twice with PBS. 20 mg of EDC (Sigma) and 25 μg of Asante Potassium Green 4, TMA+ salt (TEFLabs) were added to the beads in 1 ml of PBS, and the mixture incubated with agitation for 2 hours at room temperature, protected from light. APG4 beads were washed three times with PBS, before resuspension in 1 ml of coupling buffer containing 25 μg of Alexa Fluor 594-SE (Invitrogen), and incubation with agitation for an additional 1 hour at room temperature, protected from light. APG4/AF594 beads were washed twice with coupling buffer, and once with PBS, before final resuspension in 500 μl of PBS.

### K^+^ bead assays

Induction of APG4 fluorescence by K^+^ was tested by placing APG4/AF594 beads in assay buffer (1.54 mM NaH_2_PO_4_, 2.71 mM Na_2_HPO_4_, 157.87 mM NaCl, 5 mM dextrose, 1 mM CaCl_2_, 0.5 mM MgCl_2_) supplemented with specified concentrations of K^+^ gluconate, in a 96-well clear bottom black plate. Na^+^ gluconate supplementation was used for Na^+^ response tests. For tests with [Na^+^] less than 157.87 mM, NaCl was excluded from the assay buffer. Tests for response to pH was conducted in assay buffer with 100 mM Bis-Tris added, and pH adjusted with HCl. Each condition was tested in quadruplicate, and bottom reads of Ex. 510 nm/Em. 540 nm (APG4) and Ex. 590 nm/Em. 617 nm (AF594) taken after equilibration with a Biotek Synergy H1 microplate reader.

For intraphagosomal K^+^ bead assays, primary murine bone marrow-derived macrophages (2x10^5^/well) were seeded in a 96-well clear bottom black plate. Where needed, 100 U/ml IFNγ (PeproTech) and 10 ng/ml LPS (List Biological Laboratories) were used for activation of macrophages. 100 nM concanamycin A (AdipoGen Life Sciences) was added to the media where indicated. Macrophages were washed three times with pre-warmed assay buffer, before addition of APG4/AF594 beads (~2–5 beads/macrophage). A Biotek Synergy H1 microplate reader was used for fluorescence tracking, with four-six replicate wells/condition and bottom reads taken every two minutes for four hours. Temperature was maintained at 37°C throughout the assay.

### Mouse Mtb infections

C57BL/6J wild type mice (Jackson Laboratories) were infected intranasally with 10^3^ CFUs of Mtb (35 μl), under light anesthesia with 2% isoflurane. After sacrifice with CO_2_, the left lobe and accessory right lobe were homogenized in PBS containing 0.05% Tween 80 and serial dilutions plated on 7H10 agar plates containing 100 μg/ml cycloheximide for CFU determination. The remaining three right lobes were fixed in 4% PFA in PBS, and used for histology examination via standard hematoxylin and eosin (H&E) staining (Tufts Comparative Pathology Services). H&E stained slides were imaged on a Nikon Eclipse E400 equipped with a SPOT Insight color digital camera.

### Accession number

RNA sequencing data has been deposited in the NCBI GEO database (GSE120725).

## Supporting information

S1 Fig The K^+^ response regulon is specific and not triggered by changes in [Na^+^]. qRT-PCR of gene expression in WT Mtb grown in Na^+^-free 7H9, pH 7, compared to 7H9 with standard [Na^+^], pH 7, for 4 hours. Data are shown as means ± SD from 3 technical replicates, representative of 2 independent experiments.
(TIF)

S2 Fig *rv2390c’*::GFP does not respond to low [K^+^]. CDC1551(*rv2390c’*::GFP) was grown in standard 7H9 broth, pH 7.0 ± 250 mM NaCl, or in K^+^-free 7H9 broth, pH 7.0, supplemented with 0.01 mM KCl. Samples were taken at indicated time points, fixed, and GFP induction analyzed by FACS. Data are shown as means ± SD from 3 independent experiments.
(TIF)

S3 Fig Expression and purification of recombinant Rv0500A. Recombinant N-terminally 6x-His-tagged Rv0500A was expressed and purified from *E*. *coli* BL-21 (DE3). Coomassie Brilliant Blue R-250 stained gel shows the cleared lysate sample and the eluate fraction used in the EMSA assays.
(TIF)

S4 Fig Inhibition of H^+^-ATPase activity significantly diminishes intraphagosomal [K^+^] increase. APG4/AF594 beads were added to resting murine bone marrow-derived macrophages (MØ) in the presence of either DMSO (carrier control) or 100 nM concanamycin A, and fluorescence tracked with a microplate reader over time. Sensor beads were also added to wells containing only media, with no macrophages (“beads only, no MØs”). Data are shown as means ± SD from 4–6 wells, representative of 3 independent experiments.
(TIF)

S5 Fig Mtb Δ*ceoBC* mutant is more sensitive to low environmental [K^+^]. WT, Δ*ceoBC*, or ceoBC* (complemented mutant) Mtb carrying the K^+^ responsive *kdpF’*::GFP reporter were grown in standard 7H9 broth, pH 7.0, or in K^+^-free 7H9 broth, pH 7.0, supplemented with 0.2 mM KCl for 9 days. GFP signal in fixed samples was measured by FACS, with fold signal induction compared to the corresponding strain grown in pH 7 control media. Data are shown as means ± SD from 3 independent experiments. p-values were obtained with an unpaired t-test, * p<0.05, ** p<0.01.
(TIF)

S1 Table Genes upregulated >2-fold, p<0.05, after 4 hours exposure to K^+^-free 7H9. (XLSX)

S2 Table Genes downregulated >2-fold, p<0.05, after 4 hours exposure to K^+^-free 7H9. (XLSX)

## References

[1] Soldati T, Neyrolles O. Mycobacteria and the intraphagosomal environment: take it with a pinch of salt(s)! Traffic. 2012;13(8):1042–52. 10.1111/j.1600-0854.2012.01358.x 22462580

[2] Tan S, Sukumar N, Abramovitch RB, Parish T, Russell DG. *Mycobacterium tuberculosis* responds to chloride and pH as synergistic cues to the immune status of its host cell. PLoS Pathog. 2013;9(4):e1003282 10.1371/journal.ppat.1003282 23592993 PMC3616970

[3] Schaible UE, Sturgill-Koszycki S, Schlesinger PH, Russell DG. Cytokine activation leads to acidification and increases maturation of *Mycobacterium avium*-containing phagosomes in murine macrophages. J Immunol. 1998;160(3):1290–6. 9570546

[4] Via LE, Fratti RA, McFalone M, Pagan-Ramos E, Deretic D, Deretic V. Effects of cytokines on mycobacterial phagosome maturation. J Cell Sci. 1998;111 (Pt 7):897–905. 9490634 10.1242/jcs.111.7.897

[5] Groisman EA. The ins and outs of virulence gene expression: Mg^2+^ as a regulatory signal. BioEssays. 1998;20(1):96–101. 10.1002/(SICI)1521-1878(199801)20:1<96::AID-BIES13>3.0.CO;2-3 9504051

[6] Ratledge C, Dover LG. Iron metabolism in pathogenic bacteria. Annu Rev Microbiol. 2000;54:881–941. 10.1146/annurev.micro.54.1.881 11018148

[7] Hood MI, Mortensen BL, Moore JL, Zhang Y, Kehl-Fie TE, Sugitani N, et al Identification of an *Acinetobacter baumannii* zinc acquisition system that facilitates resistance to calprotectin-mediated zinc sequestration. PLoS Pathog. 2012;8(12):e1003068 10.1371/journal.ppat.1003068 23236280 PMC3516566

[8] Rohde KH, Abramovitch RB, Russell DG. *Mycobacterium tuberculosis* invasion of macrophages: linking bacterial gene expression to environmental cues. Cell Host Microbe. 2007;2(5):352–64. 10.1016/j.chom.2007.09.006 18005756

[9] Walters SB, Dubnau E, Kolesnikova I, Laval F, Daffe M, Smith I. The *Mycobacterium tuberculosis* PhoPR two-component system regulates genes essential for virulence and complex lipid biosynthesis. Mol Microbiol. 2006;60(2):312–30. 10.1111/j.1365-2958.2006.05102.x 16573683

[10] Vandal OH, Pierini LM, Schnappinger D, Nathan CF, Ehrt S. A membrane protein preserves intrabacterial pH in intraphagosomal *Mycobacterium tuberculosis*. Nat Med. 2008;14(8):849–54. 10.1038/nm.1795 18641659 PMC2538620

[11] World Health Organization. Global tuberculosis report. 2018. http://www.who.int/tb/publications/global_report/en/ Cited 9 January 2019.

[12] Kurachi Y, Jan LY, Lnzdunski M, editors. Potassium ion channels: Molecular structure, function, and diseases. San Diego, CA: Academic Press; 1999.

[13] Hayabuchi Y. The Action of smooth muscle cell potassium channels in the pathology of pulmonary arterial hypertension. Pediatr Cardiol. 2016;28:1–14. 10.1007/s00246-016-1491-7 27826710

[14] Zacchia M, Abategiovanni ML, Stratigis S, Capasso G. Potassium: From physiology to clinical implications. Kidney Dis (Basel). 2016;2(2):72–9. 27536695 10.1159/000446268 PMC4947686

[15] Olsen ML, Khakh BS, Skatchkov SN, Zhou M, Lee CJ, Rouach N. New insights on astrocyte ion channels: Critical for homeostasis and neuron-glia signaling. J Neuro. 2015;35(41):13827–35. 10.1523/JNEUROSCI.2603-15.2015 PMC4604221 26468182

[16] Mackenzie AB, Chirakkal H, North RA. Kv1.3 potassium channels in human alveolar macrophages. Am J Physiol Lung Cell Mol Physiol. 2003;285(4):L862–8. 10.1152/ajplung.00095.2003 12909584

[17] Bardou O, Trinh NT, Brochiero E. Molecular diversity and function of K^+^ channels in airway and alveolar epithelial cells. Am J Physiol Lung Cell Mol Physiol. 2009;296(2):L145–55. 10.1152/ajplung.90525.2008 19060226

[18] Epstein W. The roles and regulation of potassium in bacteria. Prog Nucleic Acid Res Mol Biol. 2003;75:293–320. 14604015 10.1016/s0079-6603(03)75008-9

[19] Su J, Gong H, Lai J, Main A, Lu S. The potassium transporter Trk and external potassium modulate *Salmonella enterica* protein secretion and virulence. Infect Immun. 2009;77(2):667–75. 10.1128/IAI.01027-08 19001074 PMC2632022

[20] Liu Y, Ho KK, Su J, Gong H, Chang AC, Lu S. Potassium transport of *Salmonella* is important for type III secretion and pathogenesis. Microbiology. 2013;159(Pt 8):1705–19. 10.1099/mic.0.068700-0 23728623 PMC4089031

[21] Stingl K, Brandt S, Uhlemann EM, Schmid R, Altendorf K, Zeilinger C, et al Channel-mediated potassium uptake in *Helicobacter pylori* is essential for gastric colonization. EMBO J. 2007;26(1):232–41. 10.1038/sj.emboj.7601471 17159901 PMC1782367

[22] Prindle A, Liu J, Asally M, Ly S, Garcia-Ojalvo J, Suel GM. Ion channels enable electrical communication in bacterial communities. Nature. 2015;527(7576):59–63. 10.1038/nature15709 26503040 PMC4890463

[23] Cole ST, Brosch R, Parkhill J, Garnier T, Churcher C, Harris D, et al Deciphering the biology of *Mycobacterium tuberculosis* from the complete genome sequence. Nature. 1998;393(6685):537–44. 10.1038/31159 9634230

[24] Steyn AJ, Joseph J, Bloom BR. Interaction of the sensor module of *Mycobacterium tuberculosis* H37Rv KdpD with members of the Lpr family. Mol Microbiol. 2003;47(4):1075–89. 12581360 10.1046/j.1365-2958.2003.03356.x

[25] Cholo MC, Boshoff HI, Steel HC, Cockeran R, Matlola NM, Downing KJ, et al Effects of clofazimine on potassium uptake by a Trk-deletion mutant of *Mycobacterium tuberculosis*. J Antimicrobial Chemother. 2006;57(1):79–84. 10.1093/jac/dki409 16286358

[26] Voskuil MI, Bartek IL, Visconti K, Schoolnik GK. The response of *Mycobacterium tuberculosis* to reactive oxygen and nitrogen species. Front Microbiol. 2011;2:105 10.3389/fmicb.2011.00105 21734908 PMC3119406

[27] Rodriguez GM, Voskuil MI, Gold B, Schoolnik GK, Smith I. ideR, An essential gene in *Mycobacterium tuberculosis*: role of IdeR in iron-dependent gene expression, iron metabolism, and oxidative stress response. Infect Immun. 2002;70(7):3371–81. 10.1128/IAI.70.7.3371-3381.2002 12065475 PMC128082

[28] Park HD, Guinn KM, Harrell MI, Liao R, Voskuil MI, Tompa M, et al Rv3133c/dosR is a transcription factor that mediates the hypoxic response of *Mycobacterium tuberculosis*. Mol Microbiol. 2003;48(3):833–43. 12694625 10.1046/j.1365-2958.2003.03474.x PMC1992516

[29] Laimins LA, Rhoads DB, Epstein W. Osmotic control of *kdp* operon expression in *Escherichia coli*. Proc Natl Acad Sci USA. 1981;78(1):464–8. 6787588 10.1073/pnas.78.1.464 PMC319074

[30] Frymier JS, Reed TD, Fletcher SA, Csonka LN. Characterization of transcriptional regulation of the *kdp* operon of *Salmonella typhimurium*. J Bacteriol. 1997;179(9):3061–3. 9139930 10.1128/jb.179.9.3061-3063.1997 PMC179076

[31] Price-Whelan A, Poon CK, Benson MA, Eidem TT, Roux CM, Boyd JM, et al Transcriptional profiling of *Staphylococcus aureus* during growth in 2 M NaCl leads to clarification of physiological roles for Kdp and Ktr K^+^ uptake systems. mBio. 2013;4(4):e00407–13. 10.1128/mBio.00407-13 23963175 PMC3747578

[32] Dani P, Ujaoney AK, Apte SK, Basu B. Regulation of potassium dependent ATPase (*kdp*) operon of *Deinococcus radiodurans*. PloS one. 2017;12(12):e0188998 10.1371/journal.pone.0188998 29206865 PMC5716572

[33] Ballal A, Apte SK. Differential expression of the two *kdp* operons in the nitrogen-fixing cyanobacterium *Anabaena* sp. strain L-31. Appl Environ Microbiol. 2005;71(9):5297–303. 10.1128/AEM.71.9.5297-5303.2005 16151117 PMC1214631

[34] Hatzios SK, Baer CE, Rustad TR, Siegrist MS, Pang JM, Ortega C, et al Osmosensory signaling in *Mycobacterium tuberculosis* mediated by a eukaryotic-like Ser/Thr protein kinase. Proc Natl Acad Sci USA. 2013;110(52):E5069–77. 10.1073/pnas.1321205110 24309377 PMC3876250

[35] Polarek JW, Williams G, Epstein W. The products of the *kdpDE* operon are required for expression of the Kdp ATPase of *Escherichia coli*. J Bacteriol. 1992;174(7):2145–51. 1532387 10.1128/jb.174.7.2145-2151.1992 PMC205832

[36] Rustad TR, Minch KJ, Ma S, Winkler JK, Hobbs S, Hickey M, et al Mapping and manipulating the *Mycobacterium tuberculosis* transcriptome using a transcription factor overexpression-derived regulatory network. Genome Biol. 2014;15(11):502 10.1186/s13059-014-0502-3 25380655 PMC4249609

[37] Abramovitch RB, Rohde KH, Hsu FF, Russell DG. *aprABC*: a *Mycobacterium tuberculosis* complex-specific locus that modulates pH-driven adaptation to the macrophage phagosome. Mol Microbiol. 2011;80(3):678–94. 10.1111/j.1365-2958.2011.07601.x 21401735 PMC3138066

[38] Steinberg BE, Huynh KK, Brodovitch A, Jabs S, Stauber T, Jentsch TJ, et al A cation counterflux supports lysosomal acidification. J Cell Biol. 2010;189(7):1171–86. 10.1083/jcb.200911083 20566682 PMC2894458

[39] Wagner D, Maser J, Lai B, Cai Z, Barry CE 3rd, Honer Zu Bentrup K, et al Elemental analysis of *Mycobacterium avium*-, *Mycobacterium tuberculosis*-, and *Mycobacterium smegmatis*-containing phagosomes indicates pathogen-induced microenvironments within the host cell’s endosomal system. J Immunol. 2005;174(3):1491–500. 15661908 10.4049/jimmunol.174.3.1491

[40] Reeves EP, Lu H, Jacobs HL, Messina CG, Bolsover S, Gabella G, et al Killing activity of neutrophils is mediated through activation of proteases by K^+^ flux. Nature. 2002;416(6878):291–7. 10.1038/416291a 11907569

[41] Harrison RE, Touret N, Grinstein S. Microbial killing: oxidants, proteases and ions. Curr Biol. 2002;12(10):R357–9. 12015137 10.1016/s0960-9822(02)00859-x

[42] Murphy R, DeCoursey TE. Charge compensation during the phagocyte respiratory burst. Biochim Biophys Acta. 2006;1757(8):996–1011. 10.1016/j.bbabio.2006.01.005 16483534

[43] DeCoursey TE. Voltage-gated proton channels find their dream job managing the respiratory burst in phagocytes. Physiology (Bethesda). 2010;25(1):27–40. 20134026 10.1152/physiol.00039.2009 PMC3023998

[44] MacGilvary NJ, Tan S. Fluorescent *Mycobacterium tuberculosis* reporters: illuminating host-pathogen interactions. Pathog Dis. 2018;76(3):fty017. 10.1093/femspd/fty017 PMC6086090 29718182

[45] Eriksson S, Lucchini S, Thompson A, Rhen M, Hinton JC. Unravelling the biology of macrophage infection by gene expression profiling of intracellular *Salmonella enterica*. Mol Microbiol. 2003;47(1):103–18. 12492857 10.1046/j.1365-2958.2003.03313.x

[46] Sturgill-Koszycki S, Schaible UE, Russell DG. *Mycobacterium*-containing phagosomes are accessible to early endosomes and reflect a transitional state in normal phagosome biogenesis. EMBO J. 1996;15(24):6960–8. 9003772 PMC452522

[47] Sturgill-Koszycki S, Schlesinger PH, Chakraborty P, Haddix PL, Collins HL, Fok AK, et al Lack of acidification in *Mycobacterium* phagosomes produced by exclusion of the vesicular proton-ATPase. Science. 1994;263(5147):678–81. 8303277 10.1126/science.8303277

[48] Szabo M, Wallace MI. Imaging potassium-flux through individual electropores in droplet interface bilayers. Biochim Biophys Acta. 2016;1858(3):613–7. 10.1016/j.bbamem.2015.07.009 26210300

[49] Yates RM, Hermetter A, Russell DG. The kinetics of phagosome maturation as a function of phagosome/lysosome fusion and acquisition of hydrolytic activity. Traffic. 2005;6(5):413–20. 10.1111/j.1600-0854.2005.00284.x 15813751

[50] Rhoads DB, Waters FB, Epstein W. Cation transport in *Escherichia coli*. VIII. Potassium transport mutants. J Gen Physiol. 1976;67(3):325–41. 4578 10.1085/jgp.67.3.325 PMC2214971

[51] Cholo MC, van Rensburg EJ, Anderson R. Potassium uptake systems of *Mycobacterium tuberculosis*: genomic and protein organisation and potential roles in microbial pathogenesis and chemotherapy. South Afr J Epidemiol Infect. 2008;23(4):13–6.

[52] Purdy GE, Niederweis M, Russell DG. Decreased outer membrane permeability protects mycobacteria from killing by ubiquitin-derived peptides. Mol Microbiol. 2009;73(5):844–57. 10.1111/j.1365-2958.2009.06801.x 19682257 PMC2747030

[53] Baracca A, Sgarbi G, Solaini G, Lenaz G. Rhodamine 123 as a probe of mitochondrial membrane potential: evaluation of proton flux through F(0) during ATP synthesis. Biochim Biophys Acta. 2003;1606(1–3):137–46. 14507434 10.1016/s0005-2728(03)00110-5

[54] Resnick M, Schuldiner S, Bercovier H. Bacterial membrane potential analyzed by spectrofluorocytometry. Curr Microbiol. 1985;12:183–6.

[55] Rengarajan J, Bloom BR, Rubin EJ. Genome-wide requirements for *Mycobacterium tuberculosis* adaptation and survival in macrophages. Proc Natl Acad Sci USA. 2005;102(23):8327–32. 10.1073/pnas.0503272102 15928073 PMC1142121

[56] Malm S, Tiffert Y, Micklinghoff J, Schultze S, Joost I, Weber I, et al The roles of the nitrate reductase NarGHJI, the nitrite reductase NirBD and the response regulator GlnR in nitrate assimilation of *Mycobacterium tuberculosis*. Microbiology. 2009;155(Pt 4):1332–9. 10.1099/mic.0.023275-0 19332834

[57] Williams KJ, Jenkins VA, Barton GR, Bryant WA, Krishnan N, Robertson BD. Deciphering the metabolic response of *Mycobacterium tuberculosis* to nitrogen stress. Mol Microbiol. 2015;97(6):1142–57. 10.1111/mmi.13091 26077160 PMC4950008

[58] Lopez CA, Skaar EP. The impact of dietary transition metals on host-bacterial interactions. Cell Host Microbe. 2018;23(6):737–48. 10.1016/j.chom.2018.05.008 29902439 PMC6007885

[59] Juttukonda LJ, Skaar EP. Manganese homeostasis and utilization in pathogenic bacteria. Mol Microbiol. 2015;97(2):216–28. 10.1111/mmi.13034 25898914 PMC4631260

[60] Lowry MA, Goldberg JI, Belosevic M. Induction of nitric oxide (NO) synthesis in murine macrophages requires potassium channel activity. Clin Exp Immunol. 1998;111(3):597–603. 10.1046/j.1365-2249.1998.00536.x 9528905 PMC1904888

[61] Petrilli V, Papin S, Dostert C, Mayor A, Martinon F, Tschopp J. Activation of the NALP3 inflammasome is triggered by low intracellular potassium concentration. Cell Death Differ. 2007;14(9):1583–9. 10.1038/sj.cdd.4402195 17599094

[62] Rubin EJ, Akerley BJ, Novik VN, Lampe DJ, Husson RN, Mekalanos JJ. *In vivo* transposition of mariner-based elements in enteric bacteria and mycobacteria. Proc Natl Acad Sci USA. 1999;96(4):1645–50. 9990078 10.1073/pnas.96.4.1645 PMC15546

[63] Sassetti CM, Boyd DH, Rubin EJ. Comprehensive identification of conditionally essential genes in mycobacteria. Proce Natl Acad Sci USA. 2001;98(22):12712–7. 10.1073/pnas.231275498 PMC60119 11606763

[64] Johnson BK, Scholz MB, Teal TK, Abramovitch RB. SPARTA: Simple Program for Automated reference-based bacterial RNA-seq Transcriptome Analysis. BMC Bioinformatics. 2016;17:66 10.1186/s12859-016-0923-y 26847232 PMC4743240

[65] Levdikov VM, Blagova E, Young VL, Belitsky BR, Lebedev A, Sonenshein AL, et al Structure of the branched-chain amino acid and GTP-sensing global regulator, CodY, from *Bacillus subtilis*. J Biol Chem. 2017;292(7):2714–28. 10.1074/jbc.M116.754309 28011634 PMC5314169

[66] Sukumar N, Tan S, Aldridge BB, Russell DG. Exploitation of *Mycobacterium tuberculosis* reporter strains to probe the impact of vaccination at sites of infection. PLoS Pathog. 2014;10(9):e1004394 10.1371/journal.ppat.1004394 25233380 PMC4169503

[67] Tan S, Yates RM, Russell DG. *Mycobacterium tuberculosis*: Readouts of bacterial fitness and the environment within the phagosome. Methods Mol Biol. 2017;1519:333–47. 10.1007/978-1-4939-6581-6_23 27815891 PMC5559710

[68] Yates RM, Russell DG. Real-time spectrofluorometric assays for the lumenal environment of the maturing phagosome. Methods Mol Biol. 2008;445:311–25. 10.1007/978-1-59745-157-4_20 18425459 PMC2759531

