# Supplementary figures and images for: Potassium response and homeostasis in Mycobacterium tuberculosis modulates environmental adaptation and is important for host colonization
Source: PLoS Pathog. 2019 Feb 4;15(2):e1007591. doi: 10.1371/journal.ppat.1007591 (PMC6375644; doi:10.1371/journal.ppat.1007591)

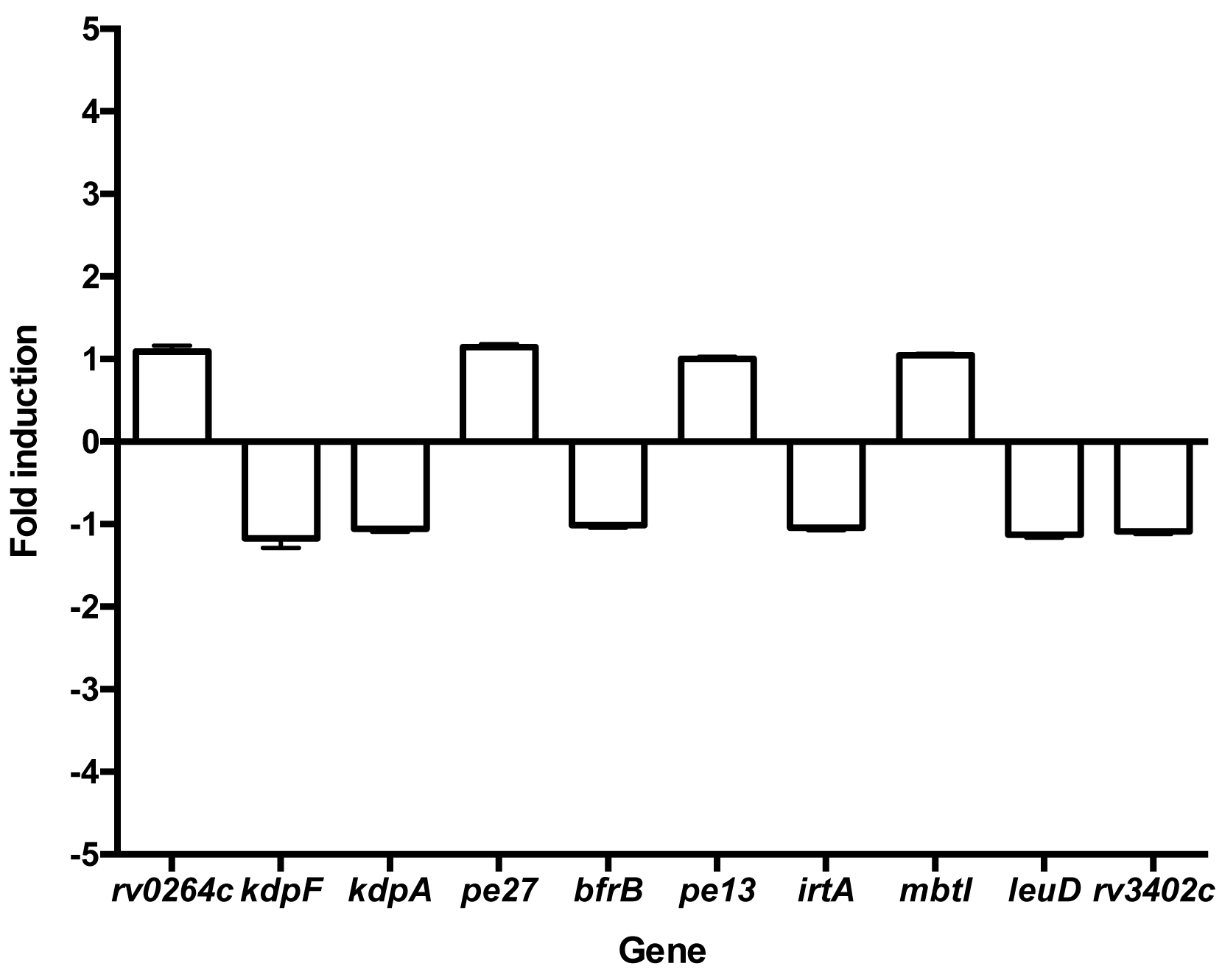

Supplement: S1 Fig — qRT-PCR of gene expression in WT Mtb grown in Na+-free 7H9, pH 7, compared to 7H9 with standard [Na+], pH 7, for 4 hours. Data are shown as means ± SD from 3 technical replicates, representative of 2 independent experiments. (TIF) [file ppat.1007591.s001.tif]

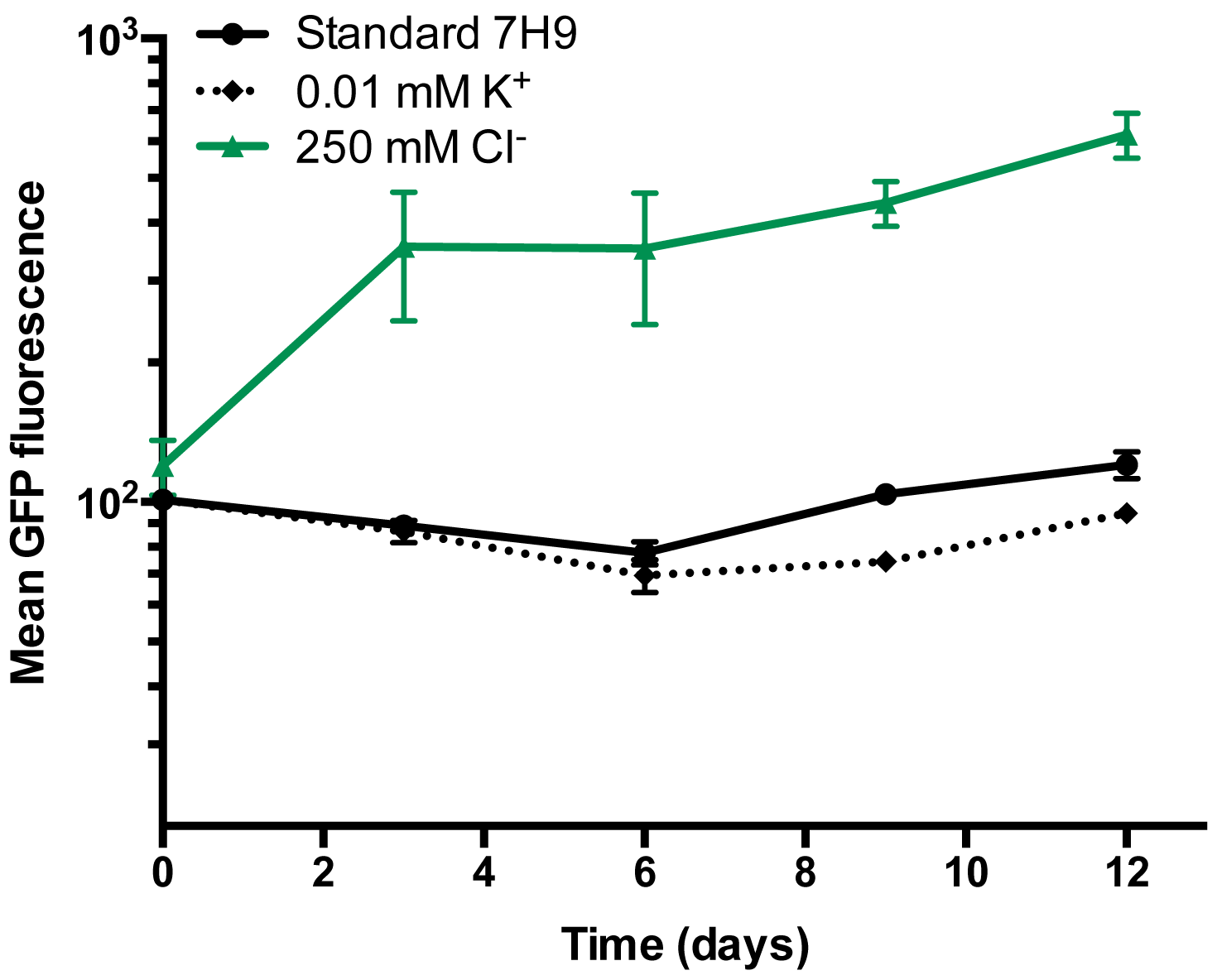

Supplement: S2 Fig — CDC1551(rv2390c’::GFP) was grown in standard 7H9 broth, pH 7.0 ± 250 mM NaCl, or in K+-free 7H9 broth, pH 7.0, supplemented with 0.01 mM KCl. Samples were taken at indicated time points, fixed, and GFP induction analyzed by FACS. Data are shown as means ± SD from 3 independent experiments. (TIF) [file ppat.1007591.s002.tif]

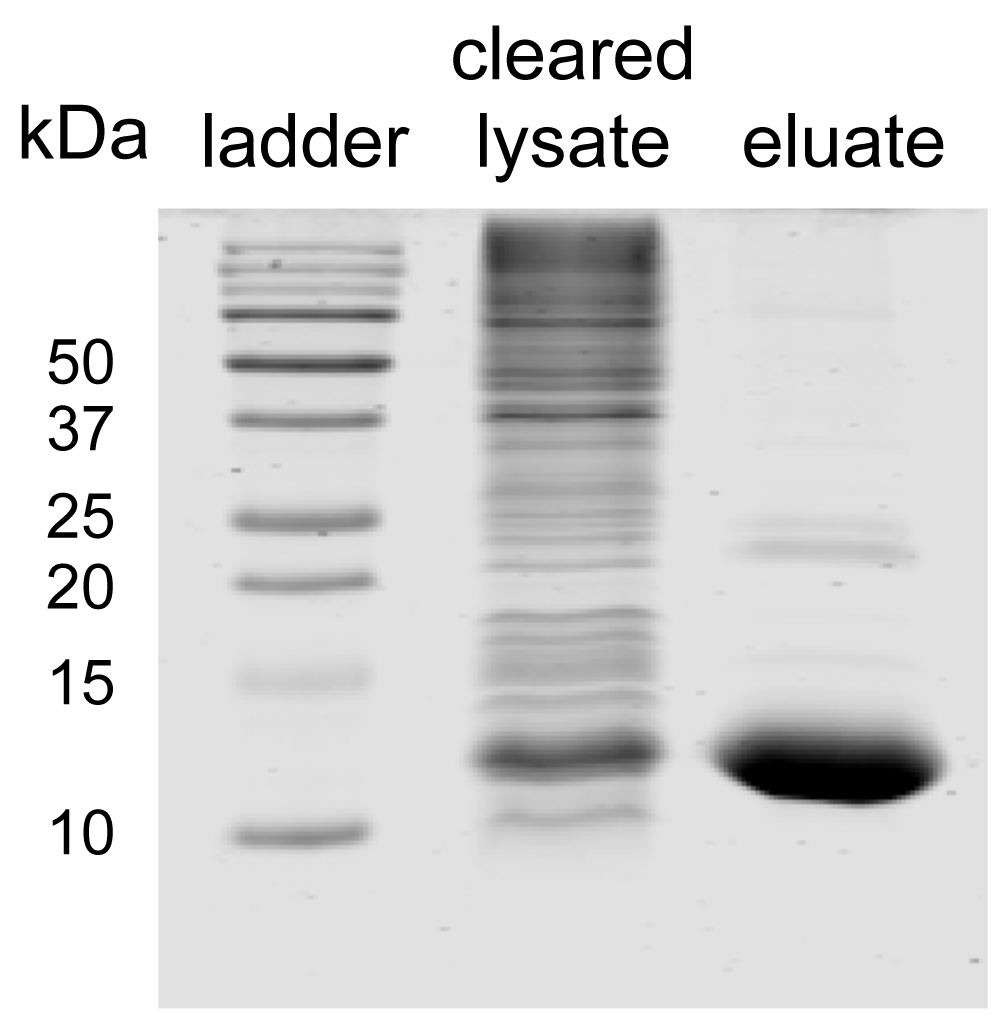

Supplement: S3 Fig — Recombinant N-terminally 6x-His-tagged Rv0500A was expressed and purified from E. coli BL-21 (DE3). Coomassie Brilliant Blue R-250 stained gel shows the cleared lysate sample and the eluate fraction used in the EMSA assays. (TIF) [file ppat.1007591.s003.tif]

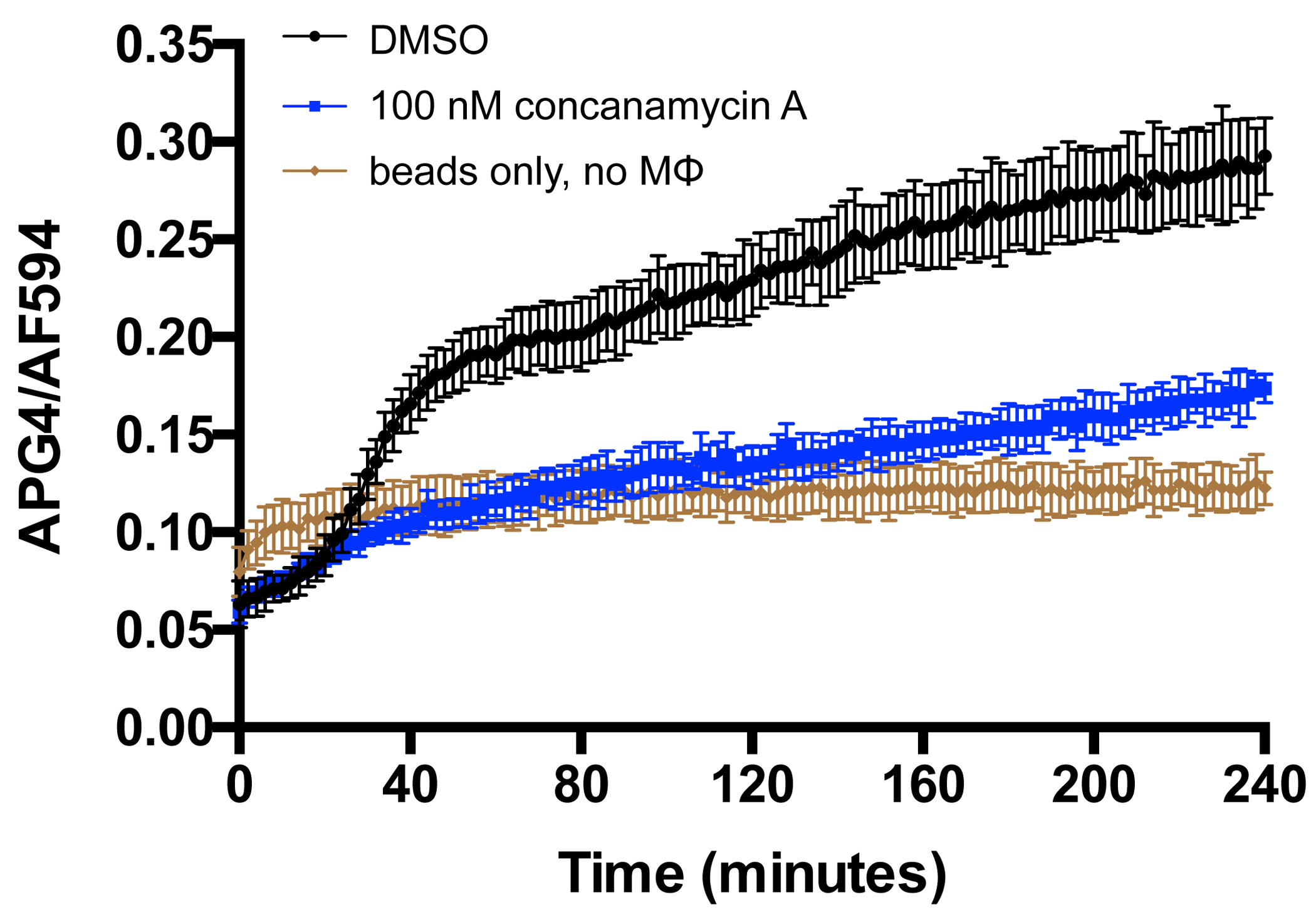

Supplement: S4 Fig — APG4/AF594 beads were added to resting murine bone marrow-derived macrophages (MØ) in the presence of either DMSO (carrier control) or 100 nM concanamycin A, and fluorescence tracked with a microplate reader over time. Sensor beads were also added to wells containing only media, with no macrophages (“beads only, no MØs”). Data are shown as means ± SD from 4–6 wells, representative of 3 independent experiments. (TIF) [file ppat.1007591.s004.tif]

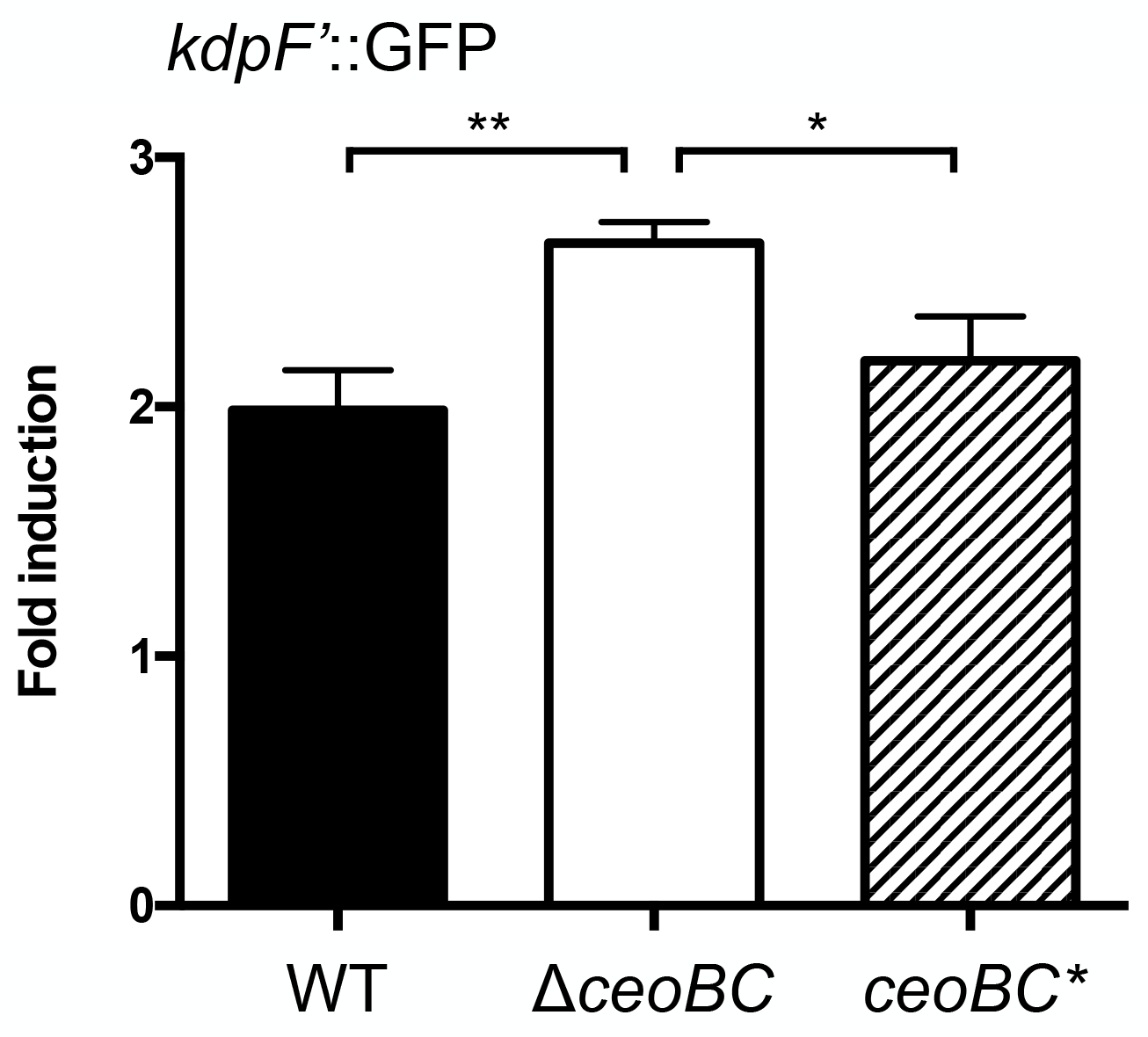

Supplement: S5 Fig — WT, ΔceoBC, or ceoBC* (complemented mutant) Mtb carrying the K+ responsive kdpF’::GFP reporter were grown in standard 7H9 broth, pH 7.0, or in K+-free 7H9 broth, pH 7.0, supplemented with 0.2 mM KCl for 9 days. GFP signal in fixed samples was measured by FACS, with fold signal induction compared to the corresponding strain grown in pH 7 control media. Data are shown as means ± SD from 3 independent experiments. p-values were obtained with an unpaired t-test, * p<0.05, ** p<0.01. (TIF) [file ppat.1007591.s005.tif]
